# Facilitators and barriers to point-of-care testing for sexually transmitted infections in low- and middle-income countries: a scoping review

**DOI:** 10.1186/s12879-022-07534-9

**Published:** 2022-06-20

**Authors:** Kevin Martin, Rhys Wenlock, Tom Roper, Ceri Butler, Jaime H. Vera

**Affiliations:** 1 Department of Clinical Research, London School of Hygiene & Tropical Medicine, London, WC1E 7HT UK; 2 Biomedical Research and Training Institute, Harare, Zimbabwe; 3 Department of Global Health and Infection, Brighton and Sussex Medical School, Brighton, UK; 4 University Hospitals Sussex NHS Foundation Trust, Brighton, UK; 5 Department of Medical Education, Brighton and Sussex Medical School, Brighton, UK

**Keywords:** Sexually transmitted infections, Point-of-care testing, Low- and middle-income countries, Scoping review

## Abstract

**Background:**

Sexually transmitted infections (STIs) in low- and middle-income countries (LMICs) are predominantly managed by syndromic management. However, most STIs are asymptomatic. These untreated STIs cause individual morbidity, and lead to high STI prevalences.

There is increasing interest in the use of point-of-care tests (POCTs) for STIs in LMICs, which could facilitate same day testing and treatment. To best utilise these tests, we must understand the facilitators and barriers to their implementation. The aim of this review is to explore how point-of-care testing for STIs has been implemented into healthcare systems in LMIC and the facilitators and barriers to doing so.

**Methods:**

A scoping review was conducted by searching MEDLINE, Embase, Emcare, CINAHL, Scopus, LILACS, the Cochrane Library, and ProQuest Dissertations and Theses for studies published between 1st January 1998 and 5th June 2020. Abstracts and full articles were screened independently by two reviewers. Studies were considered for inclusion if they assessed the acceptability, feasibility, facilitators, or barriers to implementation of point-of-care testing for chlamydia, gonorrhoea, trichomoniasis or syphilis in LMICs. Thematic analysis was used to analyse and present the facilitators and barriers to point-of-care STI testing.

**Results:**

The literature search revealed 82 articles suitable for inclusion; 44 (53.7%) from sub-Saharan Africa; 21 (25.6%) from Latin American and the Caribbean; 10 (12.2%) from East Asia and the Pacific; 6 (7.3%) from South Asia; and one (1.2%) multi-regional study. Thematic analysis revealed seven overarching themes related to the implementation of POCTs in LMICs, namely (i) Ideal test characteristics, (ii) Client factors, (iii) Healthcare provision factors, (iv) Policy, infrastructure and health system factors, (v) Training, audit, and feedback, (vi) Reaching new testing environments, and (vii) Dual testing.

**Conclusion:**

Implementation of POCTs in LMICs is complex, with many of the barriers due to wider health system weakness. In addition to pressing for broader structural change to facilitate basic healthcare delivery, these themes may also be used as a basis on which to develop future interventions. The literature was heavily skewed towards syphilis testing, and so more research needs to be conducted assessing chlamydia, gonorrhoea, and trichomoniasis testing, as well as home or self-testing.

**Supplementary Information:**

The online version contains supplementary material available at 10.1186/s12879-022-07534-9.

## Introduction

Sexually transmitted infections (STIs) cause significant morbidity worldwide [1]. Low-income countries have the highest prevalence of gonorrhoea, trichomoniasis, and syphilis [1], where contributing factors include poor access to healthcare, a lack of affordable diagnostics, and sociocultural barriers. In these settings, syndromic management is recommended, which is the provision of treatment to cover most organisms that could cause a set of signs and symptoms. However, it will therefore miss asymptomatic infections, which comprise the majority of STIs [2, 3]. Due to the limitations of syndromic management and a lack of laboratory capacity in resource-limited settings, there have been calls for the development and implementation of point-of-care tests (POCTs) for STIs as a tool to improve STI control [4, 5].

Notwithstanding the limited laboratory capacity in many low- and middle-income countries (LMICs), there are additional benefits to the provision of same day results. Laboratory testing away from the point-of-care requires multiple steps between sample collection and treatment of a positive case, with each step increasing the risk of pre-treatment loss-to-follow-up [6, 7]. Furthermore, time, cost, and infrastructural barriers may be particularly stark for patients accessing healthcare in LMIC [8]. Patients may not have the means to re-attend healthcare for their results and treatment, after their initial attendance. This was demonstrated amongst young people in Harare, Zimbabwe tested for chlamydia (CT) and gonorrhoea (NG) as part of a community-based testing strategy, where testing was laboratory-based, and results were available the following week. Of those with a positive test, 33.5% remained untreated despite attempts at active follow-up [3].

The 2021 World Health Organization (WHO) guidelines for the management of symptomatic STIs re-affirmed syndromic management as the standard of care where timely laboratory diagnosis is not available [9]. Importantly however, these guidelines introduced some guidance for the use of molecular assays for STIs with same day results, if these technologies were available. Prior to their implementation more widely, there is a need to understand the facilitators and barriers to the integration of POCTs into existing health systems, as well as the acceptability and feasibility of their use. Additionally, it is imperative to identify any gaps in the literature, to ensure resources are allocated appropriately to answer important questions in implementation research.

Multiple review papers have previously answered research questions relating to the use of POCTs for STIs in LMICs. This includes articles confirming the high sensitivities and specificities associated with syphilis rapid diagnostic tests (RDTs) [10, 11], and noting stockouts as an issue with regards to supply chain management of POCTs generally, including syphilis RDTs [12]. Systematic reviews have also demonstrated the positive impact of syphilis POCTs on syphilis screening rates in antenatal care [13, 14]. However, although some of these reviews have touched upon aspects of implementation, no review has primarily focussed on the facilitators and barriers to implementation. POCTs have the potential to significantly change the way STIs are managed in resource-limited settings. However, their implementation will only be successful if we understand how best to use them. The aim of this review is to explore how point-of-care testing for STIs has been implemented into healthcare systems in LMIC and the facilitators and barriers to doing so.

The specific objectives are to determine: (1) the facilitators and barriers to using POCTs for STIs in LMIC; (2) the acceptability and feasibility of using POCTs for STIs in LMIC; (3) How POCTs for STIs been incorporated into different models of care in LMIC; and (4) what gaps are present in the research knowledge base regarding the use of POCTs for STIs in LMIC.

## Methods

The protocol for this scoping review has been previously published [15]. The scoping review was conducted according to Joanna Briggs Institute methodology [16]. MEDLINE, Embase and Emcare (Ovid SP), CINAHL (EBSCO), Scopus, LILACS and the Cochrane Library, including the Cochrane Central Register of Controlled Trials (Wiley) were searched for articles published in English between 1st January 1998 and 5th June 2020, with the search terms (variations on and synonyms of) “sexually transmitted infections”, “point-of-care testing”, and “low- and middle-income countries”. Sources of unpublished studies and grey literature searched included ProQuest Dissertations and Theses. The full search strategy is described in Additional file 1: Appendix S1.

### Inclusion criteria

Studies were considered for inclusion if they included participants receiving or healthcare professionals providing point-of-care testing for chlamydia, gonorrhoea, trichomoniasis, and/or syphilis. Samples had to be tested at the site of sample collection. Additionally, health care and systems assessments not directly involving patients were also considered for inclusion. Human immunodeficiency virus (HIV) testing was not included in this review and so studies focusing on HIV testing alone were excluded.

Studies based in countries defined as low, lower-middle, and upper-middle economies by the World Bank, were considered for inclusion [17]. For the search strategy, the filter used for LMIC was a Cochrane filter based on the 2009 classification of countries by The World Bank [18]. This was to prevent exclusion of studies from countries that were low or middle income at the time of publication but that have since become high-income countries.

Only studies published from 1998 onwards were considered for inclusion. This was because the earliest RDTs for syphilis were developed around this time [19, 20]. Of note, rapid plasma reagin (RPR) testing was available prior to 1998, which can be used for on-site testing with provision of same day results. However, given the change in the diagnostic landscape following the development of syphilis RDTs, it was nevertheless felt appropriate to only consider studies featuring RPR after 1998.

This review was focussed on the implementation of POCTs for STIs and the facilitators and barriers to doing so. As a result, studies were only considered for inclusion if they, at least in part, assessed the acceptability, feasibility, facilitators, or barriers to implementation of point-of-care STI tests. This could be on a local, regional, national, or international level. The protocol defined facilitators as factors that promote implementation or adoption of POCTs, and barriers as factors that impede implementation or adoption of POCTs [15]. It was recognised that the definitions for feasibility and acceptability were likely to vary between studies. Feasibility was generally felt to refer to the ease and success with which POCTs were implemented logistically, whereas levels of uptake of POCTs by individuals was taken to be a surrogate for acceptability. How the POCT was implemented into a model of care was broadly split into: (i) full integration into existing services; (ii) a standalone project requiring patient consent for enrolment into the study prior to testing, but whereby treatment was prescribed based on POCT results; and (iii) a standalone project requiring patient consent for enrolment into the study prior to testing, and where POCT results required confirmatory testing prior to provision of treatment. This differentiation is necessary as interventions run by a dedicated team of study staff, not fully integrated into routine care, may not be wholly representative of how testing would work in a real-world situation.

### Types of sources

This scoping review considered primary studies with quantitative, qualitative, or mixed-methods study designs. Post-hoc changes to the protocol were made for practical reasons, due to the high number of studies suitable for inclusion. After initial abstract screening, it was decided to specifically exclude review papers, pure modelling studies, and pure economic evaluations. Similarly, abstracts for which no full text was available were also excluded.

### Study selection

Following the initial search, all identified records were collated and uploaded into EndNote X9 (Clarivate Analytics, PA, USA) and duplicates removed. The finalised list of records was then uploaded into Microsoft Excel, where titles and abstracts were independently screened by KM and RW for assessment against the inclusion criteria for the review. Each article was assigned a unique reference number, to ensure fidelity when merging spreadsheets to collate reviewer decisions and comments prior to discussion. Potentially relevant papers were retrieved in full, and the full text of selected citations were independently assessed in detail against the inclusion criteria by KM and RW. Any disagreements that arose between the two reviewers at each stage were resolved through discussion. Provision for further discussion with a third reviewer (JV) was included in the protocol, however this was not required.

The reference lists of the full text articles included in the review were screened for additional papers by KM. A list of articles was created based on article title alone, with duplicates and previously considered articles excluded. The abstracts and full texts of these articles were then assessed by KM and RW in the same manner as for the main study selection process.

### Data extraction

Data was extracted from papers included in the scoping review using a data extraction tool developed and published with the protocol [15]. The protocol specified that the data extraction tool would be modified as necessary during data extraction. The only revisions to the data extraction tool post-publication of the protocol were the addition of the data points “STI screened” and “sample type and method of sample collection”. All data was independently extracted from papers by two reviewers, including descriptive characteristics, to enhance accountability and ensure the same articles were being compared. Both reviewers then discussed this data to decide what would be written in the final table.

### Data analysis and presentation

Descriptive characteristics of research studies were presented in tabular form, including population, setting, POCT used, as well as data indicative of acceptability and feasibility.

Thematic analysis of the extracted data was used to analyse and present the facilitators and barriers to point-of-care STI testing [21]. NVivo 12 (QSR International) was used to assist with coding and theme development. Both KM and RW independently coded the notes made on the facilitators and barriers for the first ten studies. The two reviewers discussed the codes and developed a common set of initial codes. KM subsequently coded the remainder of the papers, adding new codes where necessary. An inductive approach was then used to develop themes, where were reviewed, named, and defined. Coding and themes were iteratively reviewed and refined, with regular thematic discussions between the two reviewers. Due to the nature of this project as a scoping review, coding continued past data saturation [22]. The final set of themes were presented as a narrative summary. A tree map was also produced using NVivo 12, with the size of themes and sub-themes proportional to the number of different sources coded. The Preferred Reporting Items for Systematic reviews and Meta-Analyses extension for Scoping Reviews (PRISMA-ScR) checklist is included as Additional file 2: Appendix S2.

## Results

**Fig. 1:**
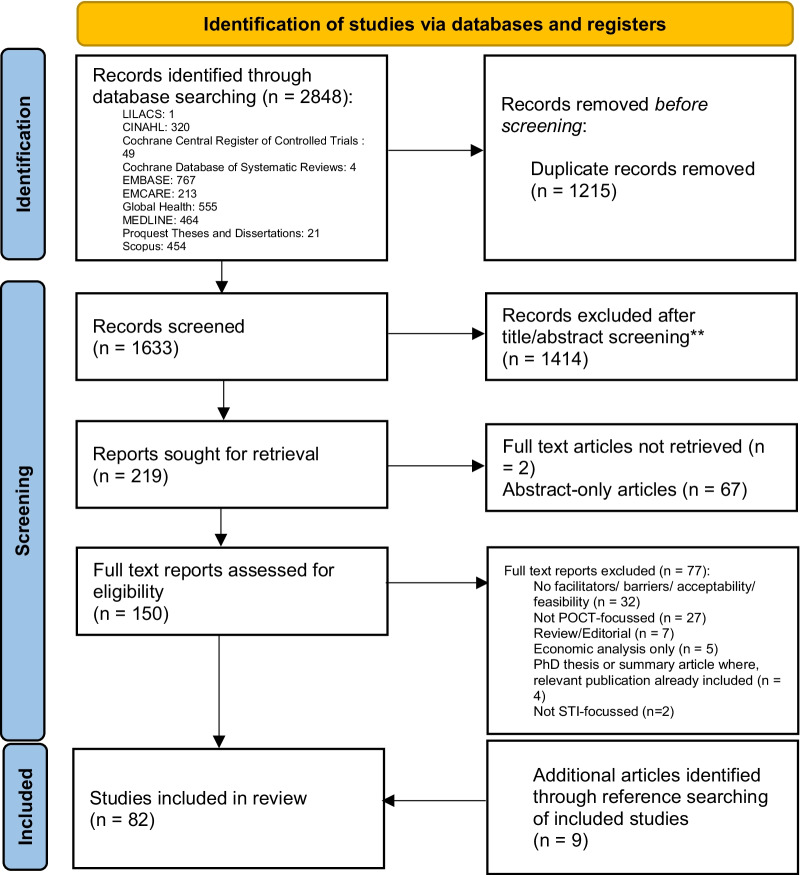
PRISMA flow diagram

The literature search performed on 5th June 2020 returned 1633 unduplicated titles and abstracts. Of these, 1414 were excluded based on title and abstract screening, leaving 219 records that were sought for retrieval. Despite assistance from the Brighton and Sussex NHS Library and Knowledge Service, the full texts for two articles were unable to be retrieved. A further 67 were abstract-only articles and were therefore excluded. There were therefore 150 full text reports assessed for eligibility, of which 73 were found eligible for inclusion. Reasons for exclusion are reported in the PRISMA diagram in Fig. 1. A further nine additional articles were identified through screening the references of the included articles, resulting in a total of 82 articles included in this review.

Data on the 82 articles is presented in Table 1, including study design, country, study population, and STI POCT used. The most common region was sub-Saharan Africa, where 44 (53.7%) studies took place, followed by 21 (25.6%) in Latin America and the Caribbean, 10 (12.2%) in East Asia and the Pacific, and 6 (7.3%) in South Asia. One (1.2%) multi-site study took place across three regions. All fingerprick and venous blood samples were healthcare worker (HCW)-collected, rather than self-testing.

**Table 1:** Table of studies included in review detailing information on study design, context, and measures of acceptability

Authors (year)	STI	Study design	Country	Setting	Study population	POCT used	Sample used & collection method	How integrated into care	Acceptability (client testing uptake)	Measures of feasibility
*Antenatal care only*										
Watson-Jones et al. (2005) [23]	TP	Multi-study data synthesis	Tanzania	ANC (mixed urban/rural)	Pregnant women	RPR	Venous blood	Integrated into routine care	–	Poor feasibility demonstrated Many facilities were failing to implement syphilis screening effectively During clinical observations of 342 ANC attenders across 9 sites, only 39% were either tested for syphilis or booked for testing Only 970/2256 eligible ANC attenders (43%) over 4 months were documented as receiving an RPR test. Of 144 (15%) who were RPR-positive, only 88 (61%) were treated and 53 (37%) had a sexual contact who attended the clinic for treatment Only 10% of 110 interviewed staff who were consulting with patients and likely to assess prenatal clients had received training in performing the RPR test
Ansbro et al. (2015) [24]	TP	Qualitative	Zambia	ANC (mixed urban/rural)	HCWs (ANC)	SD Bioline Syphilis 3.0	Fingerprick blood	Integrated into routine care	93.8% (15/16) of pilot HCWs and 62.5% (15/24) of rollout HCWs thought patients were accepting of the RST	Most HCWs agreed RSTs were successfully integrated into facility PMTCT services (16/16 pilot, 23/24 rollout HCWs)
Dassah et al. (2015) [25]	TP	Interrupted time series (before-after)	Ghana	ANC (mixed urban/rural)	Pregnant women	–	Fingerprick blood	Integrated into routine care	–	Almost all health facilities that were previously not screening pregnant women for syphilis had absolute increases in AST uptake, following the rollout However, all other facilities bar one that performed AST in 2009 had an absolute reduction in AST uptake in 2010 AST uptake of 50.0% and 33.6% in 2009 and 2010, respectively, thus leading to a much larger decrease (16.4%) in estimates of uptake following POCT rollout
Vani et al. (2015) [26]	TP	Qualitative	India	ANC (mixed urban/rural)	Key stakeholders at facility, state, and district level	–	–	Integrated into routine care	–	HCWs reported that POCT would be feasible if finger prick
Maddox et al. (2017) [27]	TP	Qualitative	Malawi	ANC (mixed urban/rural)	HCWs, laboratorians, Ministry of Health leaders and partner agency representatives	Chembio Dual Path Platform HIV-Syphilis Assay Alere Determine Syphilis TP*	Fingerprick blood	–	–	Stakeholders viewed the dual RDT as a feasible alternative to the standard tests for use in the ANC setting, although some concerns over the complexity of the dual test
Dassah et al. (2018) [28]	TP	Qualitative	Ghana	ANC (mixed urban/rural)	HCWs	Alere Determine Rapid Syphilis TP	Fingerprick blood	Integrated into routine care	–	Decentralisation of syphilis testing and provision of free syphilis testing and treatment appeared successful The main successes of the rollout programme were reported to be easy integration of syphilis screening into ANC services and its decentralisation to most public health facilities
Kanyangarara et al. (2018) [29]	TP	Secondary data analysis	DRC, Kenya, Malawi, Tanzania, Uganda, Zimbabwe, Benin, Burkina Faso, Mauritania, Senegal, Sierra Leone, Togo	ANC (mixed urban/rural)	Health facilities offering ANC	–	–	Integrated into routine care	–	Diagnostic capacity for syphilis at health facilities offering ANC varied across countries, ranging from 3% in Burkina Faso to 92% in Zimbabwe One in twelve women received ANC at a facility ready to provide syphilis detection and treatment during the first 3 months of pregnancy One in ten women received ANC during the first 3 months of pregnancy at a facility ready to provide syphilis screening
Olugbenga et al. (2018) [30]	TP	Field evaluation	Nigeria	ANC (mixed urban/rural)	Pregnant women	SD BIOLINE HIV/Syphilis Duo	Fingerprick blood	Standalone project. POCT results determine treatment	99.9% preferred dual RDT compared to single tests for HIV and syphilis	Clinic staff rated the dual RDT with an overall feasibility score 12.3/16 [Clarity of kit instruction = 2.39/3; Ease of use = 2.41/3; Ease of interpretation of results = 2.27/3; Rapidity of test results = 1.91/2; Hands-on time 1.356; Training time required = 1.95/3]
Garcia et al. (2007) [31]	TP	Implementation Study	Bolivia	ANC (mixed urban/rural)	Pregnant women	Abbott Determine Rapid Syphilis TP	Fingerprick blood	Standalone project. POCT results determine treatment	Study participants reported that they found the test highly acceptable	577/11618 (5.0%) tested positive by syphilis RDT test 93.2% (538/577) received at least a single dose of penicillin, and 81.5% (n = 470) received all three recommended doses Bolivian health policy directly influenced by study—The Ministry of Health added syphilis screening with RDTs to the national maternal health guidelines
Severe et al. (2013) [32]	TP	Time-series implementation study	Haiti	ANC (mixed urban/rural)	Pregnant women	SD Bioline Syphilis 3.0	Fingerprick blood	Integrated into routine care	Pre-POCT: 91.5% (31,810/34,776) Post-POCT: 95.9% (15,373/16,025) Post-QI intervention: 96.8% (15,916/16,435)	High rates of testing across all time periods Syphilis treatment only increased from 70.3 to 74.7% after the introduction of rapid tests (*p* = 0.28), but it improved significantly from 70.2 to 84.3% (*p* < 0.001) after the systems strengthening QI intervention
Bonawitz et al. (2015) [33]	TP	Quasi-experimental evaluation	Zambia	ANC (mixed urban/rural)	Pregnant women	SD Bioline Syphilis 3.0	Fingerprick blood	Integrated into routine care	–	Baseline: 10.3% screened for syphilis by RPR testing Midline: 67.5% screened (p < 0.001) Endline 56.3% screened (p < 0.001)
Dassah et al. (2015) [34]	TP	Case control	Ghana	ANC (mixed urban/rural)	Pregnant women	–	–	Integrated into routine care	–	–
Bocoum et al. (2017) [35]	TP	Mixed methods evaluation	Burkino Faso	ANC (mixed urban/rural)	Pregnant women	Alere Determine Syphilis TP	Fingerprick blood	Integrated into routine care	Good acceptability. Few refusals recorded	Only 39% of eligible pregnant women underwent screening, likely due to stock outs HCWs reported that the test was easy to use given its similarity to pre-existing HIV tests
Wang et al. (2018) [36]	TP	Implementation study	China	ANC (mixed urban/rural)	Pregnant women	Dual HIV/Syphilis RDT—type not specified	Fingerprick or venous blood	Standalone project. POCT results required confirmation	97.8% (1787/1828)	Feasible. Among 1787 pregnant women who received RDT tests, 98.3% (1757/1787) participants were given their test result the same day Among 1757 pregnant women received RDT testing results, 54.9% (965/1757) receiving their RDT results within 30 min, 20.7% (364/1757) received results within 30–60 min and 428 (24.4%) received results longer than 1 h later
Strasser et al. (2012) [37]	TP	Pre-post interventional study	Uganda, Zambia	ANC/PMTCT (mixed urban/rural)	Pregnant women and their male partners	SD BIOLINE Syphilis 3.0	Fingerprick or venous blood	Integrated into routine care	Zambia Baseline: 79.9% (12,761/15,967) Intervention: 95.6% (11,460/11,985) Uganda Baseline: 1.7% Intervention: 90.3% (13,131/14,540)	RST testing was easily incorporated into services with no negative effects noted on other services Zambia: Baseline—51.1% (267/523) syphilis-positive women treated. Intervention—95.2% (1000/1050) treated [958 on same day] Uganda: Intervention—5.3% (690/13,131) were positive and 103.6% (715/690) treated [Women who tested syphilis-negative but whose partner tested positive were treated, resulting in over 100% of positive cases in women treated, 708 of 715 (99.0%) of those treated received on same day No adverse effects noted on other services, and significant increases noted in percentages of pregnant women testing for HIV and receiving antiretroviral prophylaxis
Mabey et al. (2012) [38]	TP	Multi-country implementation study	Tanzania, Uganda, Zambia, China, Peru, Brazil	ANC (mixed urban/rural), community-based screening (in Amazon)	Pregnant women & sexually active population (in Amazon)	Brazil/Peru/Tanzania/Uganda/Zambia: SD Bioline Syphilis 3.0 China: Wantai anti-TP Antibody Rapid Test	Fingerprick blood	Integrated into routine care	78.1% (150,068/192,140) screened	The proportion of ANC attenders screened for syphilis increased to > 90%, and the proportion of pregnant women with syphilis who were treated the same day exceeded 90% in all countries In Brazil, HCWs in remote communities succeeded in screening 55% of the sexually active population for syphilis, exceeding the 30–40% target originally set All six countries changed their policy to recommend the use of rapid tests to provide a STAT service
Wilkinson & Sach (1998) [39]	TP	Intervention study	South Africa	ANC (mobile clinics/rural)	Pregnant women	RPR	Venous blood	Integrated into routine care	100% (398/398)	Following introduction of on-site testing, 51 of 68 women (75%) with a positive RPR test received all three doses of penicillin, compared with 22 of 45 women (49%) when testing was done in the laboratory (p = 0.004) The mean number of penicillin doses received by RPR positive women increased from 1.9 to 2.6 (p = 0.0003) Far fewer women received no or only one penicillin dose (8/68, 11.7%) when testing was done on-site than when it was laboratory-based (13/45, 28.9%; p = 0.02) With on-site testing all women received at least one penicillin dose, whereas with laboratory testing 10 (22.2%) did not receive any treatment (p = 0.0001)
Smith et al. (2015) [40]	TP	Implementation study	Guatemala	ANC (rural outreach service)	Pregnant women	SD Bioline Syphilis 3.0	Fingerprick blood	Integrated into routine care	50.3% (901/1793)	Antenatal care coverage in public healthcare services increased from 73.7 to 99.6% before and after introduction of the outreach screening program Syphilis screening increased from 49.6 to 50.3% (p = 0.87) Syphilis prevalence = 8/901 (0.89%). All syphilis cases were confirmed, treated, and their partners notified
Myer et al. (2003) [41]	TP	Cluster RCT	South Africa	ANC (rural)	Pregnant women	RPR	Venous blood	Standalone project. POCT results determine treatment	–	Mixed feasibility—No significant difference in outcomes between intervention and control clinics Nurses at busy intervention clinics frequently had difficulty in conducting the on-site test, informing women of their infection, and initiating treatment. Supply chain issues for testing materials On-site results available for 94.9% (4754/5011) eligible women The average time from the first antenatal visit to the completion of syphilis treatment was 16 days shorter for women attending intervention clinics compared to those attending control clinics (95% CI 11–21, p < 0.001) Similar proportions received no treatment (20% v 19%) and adequate treatment (64% v 69%) 3.3% of pregnancies resulted in perinatal death among women attending intervention clinics, compared with 5.1% among women attending control clinics (p = 0.31)
Bronzan et al. (2007) [42]	TP	Non-randomised trial	South Africa	ANC (rural)	Pregnant women	RPR Abbott Determine Rapid Syphilis TP	Fingerprick blood for ICS. Venous blood for on-site RPR	Standalone project but well integrated into care. POCT results determine treatment	–	Feasible. Significantly more women received at least one dose of penicillin at the intervention clinics with onsite testing Intervention clinics: 89.4% (93/104) of eligible women received at least one dose of penicillin The onsite RDT resulted in the greatest percentage of pregnant women correctly diagnosed and treated for active syphilis
Fleming et al. (2013) [43]	TP	Mixed methods	Kenya	ANC (rural)	Pregnant women	–	Fingerprick blood	Integrated into routine care	Pre-intervention: 18% (179/1586) Post-intervention: 70% (1123/1614)	Evaluation suggests RDTs are feasible for use in ANC services at low-level, rural facilities Pre-intervention: 18% (279/1586) tested During intervention: 70% (1123/1614) tested Increase in syphilis testing statistically significant at every facility At the three dispensaries, testing increased from 0 to 79% of attendees
Baker et al. (2015) [44]	TP	Mixed methods	Tanzania, Uganda	ANC (rural)	Mothers in villages and healthcare key informants	–	–	Integrated into routine care	–	Tanzania: effective coverage of syphilis screening estimated at 15% (213/1422) Uganda: effective coverage of syphilis screening estimated at 3% (88/2933)
Baker et al. (2015) [45]	TP	Cross-sectional	Tanzania	ANC (rural)	Women and ANC HCWs	–	–	Integrated into routine care	–	Estimated effective coverage of syphilis screening in Tandahimba was 12% despite near universal accessibility coverage 25% in Tandahimba and 26% in Newala received a syphilis test during ANC
De Schacht et al. (2015) [46]	TP	Quasi-experimental operational research study	Mozambique	ANC (rural)	Pregnant women, HCWs	SD Bioline Syphilis 3.0	Fingerprick blood	Integrated into routine care	–	Baseline coverage of syphilis screening: 80.8% (95%CI 65.3–96.2) After introduction of POC testing: 87.0% (95%CI 76.4–97.7); p = 0.282 Variable effect of introduction of POCTs across different facilities: Moamba and Marracuene: Significantly increased Macia: Inititally low, and remained unchanged Magude: Significantly decreased No difference in time from first ANC visit to syphilis screening—same-day testing performed both before and after introduction of POC testing
Nnko et al. (2016) [47]	TP	Qualitative	Tanzania	ANC (rural)	Pregnant women	SD Bioline Syphilis 3.0	Fingerprick blood	Integrated into routine care	100% (7954/7954)	Pre-implementation, 62% (31/50) of health facilities had the capacity to screen syphilis using RPR, however, only 22.6% (7/31) did screening Introduction of RDT doubled the number of women who attended the ANC clinics, and significantly increased the number of women who tested for syphilis at the clinics (17.9% (636/3561) vs 100% (7954/7954), p < 0.01) Proportion of pregnant women who were found with syphilis and offered treatment increased significantly (46.3% (50/108) vs 94.8% (862/909), p < 0.01) 90.1% (5719/6345) of the positive cases from September 2009 to October 2010 were tested and treated on the same day
Kuupiel et al. (2019) [48]	TP	Cross-sectional	Ghana	ANC (rural)—PHC clinics	HCWs from PHC offering ANC	–	–	Integrated into routine care	–	–
Young et al. (2018) [49]	TP	Implementation Study	Kenya	ANC (rural)—dispensaries	Pregnant women	SD Bioline Syphilis 3.0	Fingerprick blood	Integrated into routine care	Pre-POCT: 4.3% (23/529) Implementation: 97.6% (572/586)	Syphilis testing proportions increased from 4.3 to 97.6% HIV testing rates remained over 90% in all facilities before and during integrated POCT period Pre-intervention: No syphilis infections detected (0/529) During intervention: 18 syphilis infections detected (prevalence = 3.1%) Over 95% of participants received all four tests (HIV, syphilis, malaria, anaemia)
Young et al. (2019) [50]	TP	Qualitative	Kenya	ANC (rural)—dispensaries	HCWs and pregnant women	SD Bioline Syphilis 3.0	Fingerprick blood	Integrated into routine care	Improved client acceptability to POCT	–
Pai et al. (2012) [51]	TP	Cross-sectional	India	ANC (rural)—tertiary care teaching hospital	Pregnant women	Inverness Medical Determine Rapid Syphilis TP	Fingerprick blood	Standalone project. POCT results required confirmation	96.0% (1002/1066) of consented women completed testing strategy	Baseline: 8.98% (n = 90) women reported being screened for all three infections in their current pregnancy Intervention: 96% of women screened
Bique Osman et al. (2000) [52]	TP	Cluster non-randomised trial	Mozambique	ANC (suburban)	Pregnant women	RPR	Venous blood	Standalone project. POCT results determine treatment	“Very few women declined to participate”	Feasible use of RPR in clinics—5.8% false negative and 12.8% false positive compared to lab
Munkhuu et al. (2009) [53]	TP	Feasibility study	Mongolia	ANC (urban)	Pregnant women, HCWs	SD Bioline Syphilis 3.0	Fingerprick blood	Standalone project. POCT results determine treatment	100% (246/246)	Feasible
Munkhuu et al. (2009) [54]	TP	Cluster RCT	Mongolia	ANC (urban)	Pregnant women	SD Bioline Syphilis 3.0	Fingerprick blood	Standalone project. POCT results determine treatment	POCT: 99% (3849/3850) Control: 79.6% (3065/3850)	Feasible and more effective for the prevention of congenital syphilis 1st ANC visit: Control—79.6% tested. Intervention—Over 99% tested (p < 0.001) 3rd trimester: Control—62.1% tested. Intervention—99.7% tested (p < 0.001) Intervention: 73 (1.9%) and 20 (0.5%) cases of syphilis detected Control: 27(0.9%) and 2 (0.08%) cases detected Treatment: Control—89.6% (26/29) of detected cases treated. Intervention—98.9% (92/93) of detected cases treated (p = 0.02) Congenital syphilis: Control—15/3552 deliveries. Intervention—1/3632 deliveries (93.5% reduction, p = 0.002)
Gaitan-Duarte et al. (2016) [55]	TP	Cluster RCT	Colombia	ANC (urban)	Pregnant women	Arm A: SD Bioline Syphilis 3.0 & SD Bioline HIV 3.0 Arm B: SD Bioline HIV/Syphilis Duo	Fingerprick blood	Standalone project. POCT results determine treatment	Single tests: 99.8% Dual test: 99.6%	In comparison to the baseline period, syphilis testing showed an increase of 9.7% in Arm A (single HIV and syphilis RDTs) and of 6.6% in Arm B (dual HIV/syphilis RDT) Same day treatment: Single tests—69% (20/29). Dual tests—80% (16/20) Treatment at any time: Single tests—82.8% (24/29). Dual tests—100% (20/20)
Berrueta et al. (2017) [56]	TP	Cross-sectional	DRC, Zambia	ANC (urban)	Pregnant women	DRC: Alere Determine syphilis TP Zambia: SD Bioline Syphilis 3.0, RPR, or both	Fingerprick blood	Integrated into routine care	Overall: DRC: 59.7% (2479/4153) Zambia: 27.8% (5025/18066) When clinics had screening resources: DRC: 92.8% (2469/2660) Zambia: 52.0% (4761/9155)	Supplies available for screening on 78 days out of 122 in Kinshasa, and 69 days out of 129 in Lusaka Kinshasa: 59.7% (n = 2479) screened overall. 92.8% screened when test supplies available Lusaka: 27.8% (n = 5025) screened overall. 52.0% screened when tests supplies available
Nkamba et al. (2017) [57]	TP	Qualitative	DRC, Zambia	ANC (urban)	Pregnant women, clinic administrators, and HCWs	DRC: Alere Determine syphilis TP Zambia: SD Bioline Syphilis 3.0	Fingerprick blood	Standalone project. POCT results determine treatment	Testing and treatment at first ANC visit acceptable to both providers and patients	–
Althabe et al. (2019) [58]	TP	Cluster RCT	DRC, Zambia	ANC (urban)	Pregnant women	DRC: Alere Determine Syphilis TP test Zambia: SD Bioline Syphilis 3.0	Fingerprick blood	Integrated into routine care	Intervention: 99.9% Control: 93.8%	Behavioural components implemented with a compliance rate between 88 and 100% what was planned No stockouts of supplies for screening or treatment
Kasaro et al. (2019) [59]	TP	Field evaluation	Zambia	ANC (urban)	Pregnant women	SD BIOLINE HIV/Syphilis Duo Test Chembio Dual Path Platform HIV-Syphilis Assay	Fingerprick blood	Standalone project. No discussion of treatment	99.7% preferred the dual test over single tests	Both dual RDTs received high and similar feasibility scores from staff
Tinajeros et al. (2017) [60]	TP	Mixed methods	Bolivia	ANC (urban)	HCWs Clinical records of pregnant women	–	–	–	–	–
Garcia et al. (2013) [61]	TP	Feasibility study	Peru	ANC (urban/peri-urban)	Pregnant women	SD Bioline Syphilis 3.0	Fingerprick blood	Integrated into routine care	Screening coverage: 94.8% (and remained above 92% after end of implementation period)	Feasible and improved several aspects of the health system Treatment coverage 91.6%
Bocoum et al. (2015) [62]	TP	Field evaluation	Burkino Faso	ANC—Maternity ward at an urban PHC	HCWs working in the maternity ward at an urban PHC centre	1. Accu-Tell Rapid Anti-TP 2. Alere Determine syphilis TP 3. Cypress Diagnostics Syphilis quick test 4. SD Bioline Syphilis 3.0	Fingerprick blood	Standalone project. POCT results required confirmation	–	–
Pant Pai et al. (2019) [63]	TP TV	Cross-sectional	India	ANC (rural outreach service)	Pregnant women	OSOM Rapid Trichomonas Test MedMira Multiplo Rapid TP/HIV antibody test	Fingerprick blood & vaginal swab	Standalone project. POCT results required confirmation	100% (510/510) consented to testing 92% (453/491) rated testing with high satisfaction	Baseline laboratory screening rates: 42% for syphilis (214/510); 0.4% for TV (2/510) Intervention: 100% of consenting participants screened at point-of-care
Gadoth et al. (2020) [64]	CT, NG TV	Cross-sectional Field evaluation	DRC	ANC (mixed urban/rural)	Pregnant women	Xpert CT/NG Xpert TV	Clinician-collected cervical swabs	Standalone project. POCT results determine treatment	99% (366/371)	9 had invalid CT/NG tests with 3 invalid TV tests 97% of positive tests were subsequently treated
Badman et al. (2016) [65]	CT NG TV	Descriptive feasibility study	Papua New Guinea	ANC (urban)	Pregnant women	Xpert CT/NG Xpert TV	Self-collected vaginal swabs	Standalone project. POCT results determine treatment	Nearly all women who attended ANC requested to participate. 100% completion of study procedures amongst those enrolled	56% (125/222) of those who attended clinic enrolled due to limited testing facilities (one single, four-module, GeneXpert machine) combined with 20–15 new visits on a single day per week All women received their test results the same day All women with positive results had received their treatment within 1 week
Wynn (2017) [66]	CT NG TV	Cross-sectional	Botswana	ANC (urban)	Pregnant women	Xpert CT/NG Xpert TV	Self-collected vaginal swabs	Standalone project. POCT results determine treatment	85.8% (400/466) enrolled	99% of participants received results either in person (61%) on the same day as testing or by phone (39%) within a week Of 54 women with positive results: 40 received results and treated on the same day in person (74.1%), 8 received results on the same day via phone (14.8%), 5 women received delayed results and 1 woman did not receive results Overall, 52 (96%) were treated, and 77% were treated on the same day
Wynn et al. (2016) [67]	CT NG TV	Prospective cohort	Botswana	ANC (urban)	Pregnant women	Xpert CT/NG Xpert TV	Self-collected vaginal swabs	Standalone project. POCT results determine treatment	89% (200/225)	Feasible. 100% of consenting women successfully tested for CT, NG, and TV, and given their results One sample re-tested as temperature of the Xpert machine was above the threshold 72% (n = 143) received their results in person on the day of testing 29% (n = 57) contacted by telephone, on the same day, after leaving the clinic as could not wait for their STI results 100% of women who tested positive for an STI were successfully treated, most immediately (80%)
Morikawa et al. (2018) [68]	CT NG TV	Field evaluation	South Africa	ANC for women living with HIV—PHC clinics (urban/peri-urban)	Pregnant women living with HIV	Xpert CT/NG Xpert TV	Self-collected vaginal swabs	Integrated into routine care	97.3% (430/442)	91.9% (159/173) of those with a positive STI test result received same-day results and treatment 100% of women with an STI were treated within 7 days
*Both antenatal care & non-antenatal settings*										
Bristow et al. (2017) [69]	TP	Conjoint analysis	Haiti	Health centres offering STI/HIV testing and ANC	Men and women seeking STI/HIV testing or ANC at health centres	–	–	–	–	–
Laksanasopin et al. (2015) [70]	TP	Field evaluation	Rwanda	PMTCT clinics or VCT centres (3 urban community-level health centres)	PMTCT or VCT clinic attendees	Smartphone dongle with a triplexed immunoassay (HIV antibody, treponemal and non-treponemal antibodies for syphilis)	Fingerprick blood	Standalone project. No discussion of treatment	97% of patients preferred the dongle compared to laboratory-based tests	HCWs reported good feasibility
Fehler & Ballard (1998) [71]	TP	Cross-sectional pilot	South Africa	STD clinic (urban) & PHC clinic providing ANC (informal settlement)	Pregnant women & symptomatic STI clinic attendees	RPR	Venous blood	Integrated into routine care	–	Feasible Before on-site screening: 73% (29/40) of reactive RPR tests at STD clinic and 50% (10/20) at ANC clinic treated for syphilis. Presumed that all those with positive RPR on-site received treatment although not explicitly stated
Withers et al. (2019) [72]	TP	Field evaluation	Vietnam	STI clinic (for MSM) and ANC	MSM and pregnant women	SD BIOLINE HIV/Syphilis Duo	Fingerprick blood	Standalone project. POCT results required confirmation	–	–
Flores et al. (2015) [73]	TP	Quasi-experimental time-series study	Peru	Reference hospital (peri-urban)	Hospital users seeking HIV/TP testing or pregnant women seeking ANC	SD Bioline Syphilis 3.0	Fingerprick blood	Integrated into routine care	52% clients completely satisfied and 48% satisfied with point-of-care testing	Baseline period: 61.0% of pregnant women tested for syphilis received timely results (within 45 min). Clients that did not receive their results within the 45-min norm, obtained their results after a minimum of 24 h, and often following days and weeks Intervention period: 100% of pregnant women other key populations received timely results with POCTs
Marks et al. (2018) [74]	TP & Yaws	Qualitative	Solomon Islands	Outpatient and ANC departments of a district hospital and four rural health clinics	HCWs and clinic patients	Chembio Dual Path Platform Syphilis Screen and Confirm test kit	Fingerprick blood	Integrated into routine care	–	–
*Non-antenatal settings*										
Gupte et al. (2011) [75]	TP	Cross-sectional	India	Fixed, satellite and mobile clinics offering services for sex workers	Sex workers (female, male and transgender)	Qualpro Syphicheck-WB	Fingerprick blood	Standalone project. POCT results required confirmation	63.1% (19,809/31,395)	Introduction of rapid test improved feasibility of providing syphilis testing in all clinical settings, including mobile venues. Among the 19,809 SWs who accepted rapid syphilis testing, 598 tested positive (3.0% prevalence of lifetime syphilis infection). Among those screened with a positive rapid test, two-thirds accepted a confirmatory RPR test, with a reactivity rate of 85.3% (any titer) and 40.5% for active syphilis (RPR titer >/= 1:8)
Chen et al. (2012) [76]	TP	Cross-sectional	China	FSW outreach services at sex work venues	FSW	Wantai anti-TP Antibody Rapid Test	Fingerprick blood	Integrated into existing FSW outreach services—Clients referred to clinics for further diagnosis/treatment	95.0% (2670/2812)	Feasible to integrate into FSW outreach services RDT results: 182/2670 (6.8%) had a positive result 136/182 (74.7%) of FSW with positive results were willing to attend a clinic for further confirmatory testing and treatment
Campos et al. (2006) [77]	TP	Field evaluation	Peru	Mobile outreach to commercial sex venues	FSW	Alere Determine syphilis TP	Fingerprick blood	Standalone project. POCT results determine treatment	97.4% (3586/3682)	0.3% invalid test rate. 87% POCT positives subsequently went onto get treatment and 64% completed the three-dose regimen. Relatively easy to use operationally and to incorporate into existing services
Parthasarathy et al. (2013) [78]	TP	Retrospective analysis	India	STI clinic	FSW, MSM, IVDU	Immunochromatographic strip test & RPR	Fingerprick blood	Integrated into STI programme	–	Avahan experience demonstrated that syphilis screening could be effectively integrated into a large-scale HIV prevention programme The proportion of RDT used for screening increased from 7.4% in 2007 to 77.0% (p < 0.001) in 2009, replacing RPR as the predominant screening test. During the same period, the syphilis screening rates among clinic attendees increased from 9.0 to 21.6% (p < 0.001)
Mishra et al. (2010) [79]	TP	Field evaluation	India	STI clinic (fixed and mobile site camps)	FSW	Qualpro Syphicheck-WB	Fingerprick blood	Integrated into syphilis screening programme	POC overall: 33.4% (1627/4871) POC, first-time attendees: 26.9% (1117/4157) Standard protocol, first-time attendees: 18.9% (1017/5391)	Treatment completion: Off-site RPR = 44.8%. POC screening = 68.3% (p = 0.003) POC—97.5% (115/118) of FSWs who tested positive received same-day treatment This corresponding to at least one dose of treatment in 68.3% of women with active syphilis, as determined by the reference laboratory
Sabido et al. (2009) [80]	TP	Field evaluation	Brazil	STI clinic (urban)	STI clinic attendees including FSW, male clients of FSW, and other patients living or working in Manaus harbour area. HCWs and laboratory technicians	Omega Diagnostics VisiTect Syphilis	Fingerprick blood	Standalone project. POCT results determine treatment	52% of respondents stated that they would choose the conventional test over POCT	Operationally appropriate. Results of the time-flow analysis conducted among 84 patients showed that, excluding time spent receiving treatment for 7 (8.3%) patients, average time spent at the clinic was 51 min (SD 32)
Bristow et al. (2018) [81]	TP	Conjoint analysis	Peru	STI clinic (urban) and a gay men’s community health centre	MSM, TGW	–	–	–	–	–
Benzaken et al. (2008) [82]	TP	Field evaluation	Brazil	STI clinic (urban) & outreach clinic offering services near red-light district	STI clinic attendees including male and female sex workers and sex worker clients	Omega Diagnostics VisiTect Syphilis	Fingerprick blood	Standalone project. POCT results determine treatment	Reported as largely acceptable	–
Bien et al. (2015) [83]	TP	Qualitative	China	Urban community-based locations: (1) A local VCT site, (2) A local community-based organisation with ties to MSM community	MSM	–	–	–	–	–
Lipsitz et al. (2014) [84]	TP	Cross-sectional	Peru	Mobile testing unit (outreach)	MSM, TGW (although open to all visitors)	Inverness Medical Determine Rapid Syphilis TP	Fingerprick blood	Standalone project. POCT results required confirmation. (Positive results referred)	–	–
Allan-Blitz et al. (2019) [85]	TP	Cross-sectional	Peru	Outreach to MSM/TGW venues	MSM, TGW	SD BIOLINE HIV/Syphilis Duo	Fingerprick blood	Standalone project. POCT results required confirmation	303/585 (51.8%)	–
Pinto et al. (2014) [86]	TP	Cross-sectional	Brazil	Social support services (including shelters, hostels, homes and temporary charitable housings)	Homeless population with assisted social services	Omega Diagnostics VisiTect Syphilis	Fingerprick blood	Standalone project. POCT results determine treatment	86.6% (1405/2110)	All individuals with a positive RDT result agreed to start treatment right away, and, through the guidance of the multidisciplinary team, they were referred to health units to complete the treatment and for monitoring of the cure process
Hall et al. (2020) [87]	TP	Mixed methods	Macao, China	Community-based NGO study field site	Female Filipino migrant domestic workers	ABON Syphilis Ultra Rapid Test Device	Fingerprick blood	Standalone project. POCT results required confirmation (positive results referred)	85.3% (1164/1363)	–
Benzaken et al. (2007) [88]	TP	Field evaluation	Brazil	STI clinic (urban)	Patients self-presenting to a STI clinic	1. Qualpro Syphicheck-WB 2. SD Bioline Syphilis 3.0 3. Abbott Determine Rapid Syphilis TP 4. Omega Diagnostics VisiTect Syphilis	Venous blood	Standalone project. POCT results required confirmation	–	High reproducibility between clinic and lab teams (kappa > 0.9 across all test scenarios)
Gallo Vaulet et al. (2018) [89]	TP	Field evaluation	Argentina	STI clinic (urban)	Patients self-presenting to a STI clinic (50.2% MSM)	Alere Determine syphilis TP	Fingerprick blood	Integrated into routine care	31.1% (587/1887)	–
Ribeiro et al. (2015) [90]	TP	Cross-sectional	Brazil	Home	Residents in urban area of Amazonas state	SD Bioline Syphilis 3.0	Fingerprick blood	Standalone project. POCT results required confirmation	85.6% (1501/1752)	Eleven HCW evaluated four sample tubes for HIV and four DTS for syphilis. Of those, three (27.3%) interpreted at least one test result incorrectly for syphilis Overall, 40/44 (90.9%) of the syphilis readings on dried test spots were correct 3 syphilis dried test spots (6.8%) were falsely reported as negatives Nurses reported all the incorrect syphilis readings (as opposed to nurse practitioners)
Mark et al. (2017) [91]	TP	Observational cohort study	Kenya	Home	Male partners of pregnant women	SD Bioline Syphilis 3.0	Fingerprick blood	Standalone project. POCT results determine treatment	During partner’s pregnancy: 93% (74/80) 6 months post-partum: 98% (226/230)	Feasible and no effect on HIV testing 96% (151/158) accepted HIV testing prior to syphilis test introduction 95% (70/74) accepted HIV testing when both HIV and syphilis testing were offered
Ruffinen et al. (2015) [92]	TP	Implementation study	Brazil	PHC	HCWs involved in POC testing	SD Bioline Syphilis 3.0	Fingerprick blood	Integrated into routine care	HCWs reported that the acceptance of rapid testing was excellent or good at all ten polo bases	3 months after the introduction, 25.9% (6473/25,322) screened in Alto Solimões DSEI (11 polo bases) Syphilis prevalence = 2.5% (165/6473) 86.7% of all syphilis-positive individuals started treatment following the rapid test and that all were referred for confirmatory testing. Failure to immediately treat syphilis-positive individuals was primarily caused by a lack of benzathine benzylpenicillin at four of the ten polo bases
Mashamba-Thompson et al. (2018) [93]	TP	Cross-sectional	South Africa	Rural PHC	PHC HCWs (operations managers, PHC specialist nurses, staff nurses)	–	–	Integrated into routine care	–	Only 0.5% of rural PHCs have currently accessible, available and in use syphilis tests
Smit et al. (2013) [94]	TP	Cross-sectional	Tanzania	Demographic surveillance study	Participants of community-based study (representative of population)	SD Bioline Syphilis 3.0	Fingerprick blood	Integrated into larger demographic surveillance study	–	–
West et al. (2002) [95]	TP	Cross-sectional	The Gambia	Rural community—field laboratory set up in 20 villages for a reproductive health survey	Women of reproductive age aged 15–54	RPR Quorum Diagnostics RST	Venous blood	Standalone project. No discussion of treatment	–	97.7% (1295/1325) RPR tested in the field (field screening in these 30 women not carried out owing to logistical difficulties, either generator or equipment failure or lack of consumables in the field laboratory)
Parkes-Ratanshi et al. (2019) [96]	TP	E-mail survey	Uganda	Mixed public/private sector facilities	HCWs	–	–	–	–	–
Verwijs et al. (2019) [97]	CT NG TV TP	Cross-sectional	Rwanda	Research clinic	Women aged 18 years or older at risk of acquiring STIs	Xpert CT/NG OSOM Rapid Trichomonas Test Alere Determine syphilis TP	Clinician-collected cervical swabs, Fingerprick blood	Standalone project. POCT results determine treatment. WISH algorithms used to determine who screened	100% (705/705) of enrolled participants accepted STI testing. 15.3% rejected HIV testing. 86.9% (344/396) chose to wait for the results. 100% clients who completed a satisfaction survey liked all testing procedures	Staff and participants considered point-of-care testing feasible and acceptable with tests easy to perform and interpret Prevalences: CT = 8.5% (60/705). NG = 7·1% (50/705). TV = 16.1% (111/690) WISH algorithms: CT sensitivity 71·7%, specificity 100%, NG sensitivity 76·0%, specificity 100%, and TV sensitivity 68·5%, specificity 97·4%
Garrett et al. (2018) [98]	CT NG TV	Prospective cohort pilot study	South Africa	Large urban public healthcare clinic	HIV negative women presenting for STI care	Xpert CT/NG OSOM Rapid Trichomonas Test	Clinician-collected blind vaginal swabs	Standalone project. POCT results determine treatment	–	23.6% (63/267) were diagnosed with at least one of CT/NG/TV
Stime et al. (2018) [99]	CT NG TV	Mixed methods (time in motion study, qualitative)	South Africa	Large urban public healthcare clinic	STI clinic attendees	Xpert CT/NG OSOM Rapid Trichomonas Test	Clinician-collected blind vaginal swabs	Standalone project. POCT results determine treatment	–	Syndromic management (n = 39): mean total visit duration = 2:05 and the mean clinical appointment duration was seven minutes STI POCT (n = 9): mean total visit duration = 4:26. Mean additional visit time of 2:49 of which the longest step was running the GeneXpert samples (2:12). While the clinical visit accounted for 64% of the total time, much of this time was spent waiting for results Staff in favour of expanding POCT
Badman et al. (2019) [100]	CT NG	Diagnostic evaluation	Papua New Guinea	Survey (urban)	FSW, MSM, TGW	Xpert CT/NG	Self-collected anorectal swabs	Standalone project. POCT results determine treatment	99.95% (2134/2135)	98% (2095/2134) valid test results—144 (6.7%) invalid at first test, of which 105 (72.9%) generated a valid test on repeating testing
Jones et al. (2007) [101]	TV	RCT	South Africa	Home	Women aged 14–25	XenoStrip TV test^#^	Self-collected vaginal swabs	Standalone project. POCT results determine treatment	97% of women at the 6-week interview who successfully self-sampled reported that they would self-sample in the future	87% (146/168) test kits were received at the clinic from women who reported mailing them 96% contained the self-collected swab for PCR testing, 79% contained the used TV test strip, and 77% the self-administered questionnaire Most women reported that self-sampling was easy or very easy, but more women in the clinic group reported this than women in the home group. 95% of these women reported being able to read the results, and 92% of the recorded results matched the reading by clinic staff. Almost all clinic women (98%) who came for their appointment were rated as finding it easy or very easy to perform the rapid test on their own. All women who tested positive were treated in both groups
Lippman et al. (2007) [102]	TV	RCT	Brazil	Home	Women aged 18–40 years recruited from the general clinic population and from the clinic catchment area	XenoStrip TV test	Self-collected vaginal swabs	Standalone project. POCT results required confirmation	96% (n = 787) were comfortable collecting their own vaginal sample and found self-collection easy	Overall, home-based testing was feasible: 94% of home group participants were able to complete collection and self-testing at home on their first attempt 80% of women in the home group returned samples to the study clinic within 2 weeks of enrolment compared with 76% of women presenting for screening in the clinic group (one-sided p = 0.06) Following a reminder phone call or letter for non-responders at 2 weeks, a slightly higher proportion of home group participants responded before the 6-week follow-up visit (93%—381/410) as compared with clinic group response (89% (359/403) (one-sided p = 0.03)
Benzaken et al. (2006) [103]	NG	Field evaluation	Brazil	STI clinic (urban)	Women with vaginal discharge or referred by a partner with urethral discharge	NGThermo Biostar	Clinician-collected cervical swabs	Standalone project. No discussion of treatment	–	Three of the four staff members were able to obtain results within 30 min of receiving the specimen
Yin et al. (2006) [104]	CT	Field evaluation	China	STI clinics, female re-education centres and sex entertainment venues (urban)	Women at risk of chlamydia infection	Clearview Chlamydia MF	Clinician collected vaginal and cervical swabs	Standalone project. No discussion of treatment	–	Excellent agreements between the results read by two independent staff for either vaginal or cervical specimens in different study sites (k statistics 0.94–1.00 vaginal specimens and 0.96–1.00 for cervical specimens; all p values were < 0.001)

*ANC* antenatal care; *CT Chlamydia trachomatis*; *DRC* Democratic Republic of the Congo; *FSW* female sex worker; *HCW* Healthcare worker; *IVDU* intravenous drug user; *MSM* men who have sex with men; *NG Neisseria gonorrhoeae*; *NGO* Non-governmental Organisation; *PHC* primary healthcare; *POCT* point-of-care test; *RCT* randomised controlled trial; *RPR* rapid plasma reagin; *SD* standard diagnostics (company); *STI* sexually transmitted infection; *TGW* transgender women; *TV Trichomonas vaginalis*; *TP Treponema pallidum*; *VCT* voluntary counselling and testing

*Alere, formerly known as Inverness Medical Innovations, Inc, until 2010, acquired the Determine line of rapid tests from Abbott laboratories in 2005. Alere was subsequently acquired by Abbott in 2017

^#^The Xenostrip TV test was acquired by Genzyme and sold under the brand OSOM. Sekisui acquired Genzyme’s diagnostics products business in 2011, and continues to sell the test as the OSOM Trichomonas rapid test

The majority (85.4% = 70/82) of articles featured syphilis testing, whereas only 11 (13.4%) included trichomoniasis (TV) testing, 10 (12.2%) included CT testing, and 10 (12.2%) included NG testing.

Testing was provided in antenatal care only in 46 (56.1%) articles; 15 in urban/sub-urban regions; 14 in rural areas; and 17 covering both urban and rural areas. 14 (17.1%) papers focussed testing on key and vulnerable populations including sex workers and their clients, men who have sex with men (MSM), transgender women (TGW), migrant domestic workers, and homeless individuals. Other testing locations included STI/HIV services (7/82 = 8.5%), home testing (4/82 = 4.9%), primary healthcare (2/82 = 2.4%), and as part of demographic surveillance surveys (2/82 = 2.4%). One (1.2%) paper featured a survey of healthcare professionals across a range of testing locations [96]. A further six (7.3%) articles included testing for both pregnant women and another population [69–74].

### Acceptability, feasibility and integration into care of point-of-care testing

Measures of acceptability for studies included in the review are reported in Table 1. Generally, high levels of uptake of point-of-care testing were noted. Importantly, in some of the studies with lower levels of uptake there was evidence of ‘overlap’ with feasibility whereby testing was not always offered due to stock or staffing issues [40], or where the requirement for venepuncture for confirmatory testing was noted as a bigger deterrent than the POCT [79]. Lower levels of uptake were also noted to cluster in studies featuring marginalised populations including MSM, TGW and sex workers [75, 79, 85, 89].

How feasibility was measured or could be interpreted varied significantly between studies (see Table 1 for full list of feasibility measures). Overall, POCTs were felt to be feasible and were implemented successfully.

Table 1 also describes whether POCTs were fully integrated into routine care, or whether they were standalone projects. Importantly, only one study investigated the use of an algorithm to allocate testing. Verwijs et al. developed an algorithm whereby they tested all participants for TV, but only tested for CT, NG, and syphilis, if they had a “positive risk score” [97]. Their algorithm had sensitivities ranging between 68.5 and 76.0% and specificities ranging between 97.4 and 100.0% for CT, NG, TV and syphilis, which was noted to be far superior to the WHO syndromic management algorithm.

### Thematic analysis of facilitators and barriers to point-of-care testing for STIs in LMIC

**Fig. 2:**
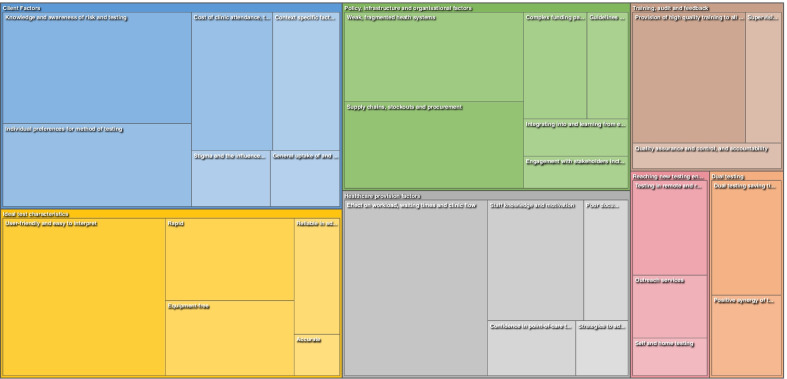
Tree map showing thematic analysis of facilitators and barriers to point-of-care testing for STIs in LMIC (the area associated with individual themes and sub-themes is proportional to the number of different sources coded)

Of the 82 studies included in the review, data on both facilitators and barriers, on facilitators alone, and on barriers alone, was able to be extracted from 53, 12, and 15 articles, respectively. Thematic analysis of the extracted facilitators and barriers revealed seven over-arching themes related to the provision of point-of-care testing for STIs in LMIC. These are (i) Ideal test characteristics, (ii) Client factors, (iii) Healthcare provision factors, (iv) Policy, infrastructure and health system factors, (v) Training, audit, and feedback, (vi) Reaching new testing environments, and (vii) Dual testing. Figure 2 demonstrates a tree map showing these themes and their sub-themes.

### Ideal test characteristics

#### Accurate

In two studies assessing test attributes that affected willingness to test by clients, no potential for a false positive result had the largest impact [72, 81]. Additionally, health workers valued accurate tests, with concordance between POCTs and laboratory tests reinforcing their confidence in them [74]

#### Equipment-free

Requirements for an electrical supply, sufficient space, and ongoing maintenance needs are important barriers relevant to testing using the GeneXpert platform for CT, NG and TV [64, 67, 68, 99, 100]. The RPR test was also felt to be overly complex for on-site testing [41], with equipment or generator failure sometimes leading to an inability to complete screening [95].

Conversely, the lack of a need for an external power source or any form of laboratory infrastructure was often cited as one of the main advantages for simpler tests such as syphilis RDTs [47, 70, 92, 94].

Preferences were also noted for pre-prepared testing kits [58], that did not require any additional parts in order to conduct the test [62], as well as algorithms to simplify the whole testing process [47]

#### Rapid

A rapid turnaround time was noted from both clients and healthcare providers across a large number of studies as a major facilitator to point-of-care testing [36, 42, 47, 53, 59, 61, 62, 70, 72–75, 80, 81, 83, 88, 89, 94, 96, 104]. This was noted to reduce anxiety by reducing the wait time for clients [83]. Reduced analytic time will also increase potential testing capacity; in a study by Badman et al., having access to only a single four-module GeneXpert machine with an analytic time of ninety minutes, resulted in only 56% of pregnant women being enrolled due to limited analytic capacity [65].

#### Reliable in adverse conditions

In LMIC, testing may need to be conducted in challenging conditions including field sites with high temperatures, dusty conditions, and inadequate lighting [59, 77, 95]. As a result, tests must be robust and useable in these settings, returning low numbers of invalid tests [77]. Similarly, the need for appropriate storage conditions was a potential barrier to the use of POCTs [23, 26, 92, 94]. As such, test kits that are more resilient to fluctuating, extreme environmental conditions may be better suited to use in LMIC.

#### User-friendly and easy to interpret

User-friendliness of POCTs was a commonly noted facilitator to their use [24, 35, 42, 50, 59, 62, 64, 70, 74, 80, 82, 88, 92, 96, 97, 101–104]. Specifically, clear instructions [104], ease of interpretation [70, 102, 104] and minimal training requirement [47, 59, 64, 65, 70, 89, 92, 104] were described. Additionally, the similarity to other rapid tests, such as those for HIV or malaria, was felt to aid uptake and implementation by HCWs [35, 47, 57, 74].

Challenges to conducting testing including difficulties with extracting blood for fingerprick testing [42, 50, 51, 74], results interpretation [24, 27, 30, 42, 71, 80, 90], visual difficulties of healthcare professionals affecting reading of results [59, 61], and errors as a result of mistakes with timing and volume of buffer [74]. Importantly, the more complex the test, the more stringent the training requirements will be, which is a potential barrier to its use [27, 68, 71, 100].

### Client factors

#### Cost of clinic attendance, testing and treatment

Clients noted multiple barriers to reaching clinics and to undertaking testing and treatment. These included the actual cost of testing and treatment for the client or their partner, as well as transport costs to attend healthcare facilities [43, 50, 69, 72, 81]. Young et al. noted that sometimes pregnant women would not attend referral services, which were often further away, due to additional travel costs [50].

As well as the direct financial costs associated with accessing healthcare, indirect financial and non-financial costs may also be present. Clients often noted a “lack of time” for testing, particularly where additional or long waiting times were present [32, 40, 66, 67, 85–87]. This may be a particular barrier for clients who have competing priorities such as the need to go to work [87]. This is further exacerbated when travelling long distances is required to access healthcare [31, 57, 65, 85, 102].

#### Knowledge and awareness of risk and testing

Clients’ awareness of their risk of infection and the importance of testing, as well as their trust in the testing of process, were important factors governing individuals’ willingness to be tested. Low perception of risk was often noted as a reason for test refusal [38, 47, 87, 91], as was a lack of interest or awareness in the need for testing [32, 60, 67, 85, 86, 91, 92].

Furthermore, misconceptions existed surrounding both the consequences of infections and the testing process itself. These included concerns about clandestine HIV testing [79], mistaking rapid syphilis testing for HIV testing [47], beliefs that testing for syphilis involved a urine sample or vaginal examination [57], and limited knowledge of the consequences of syphilis, particularly for babies [47, 57]. Additionally, worries about positive results acted as barriers to testing uptake [38, 40, 87, 92]. However, three of the four studies where this was noted involved dual HIV/syphilis testing and so the fear and stigma surrounding HIV may have had an impact.

Conversely, where clients were engaged in the testing process, there was often noted to be a willingness to wait for their results, and an appreciation of same day results [30, 47, 53, 57, 63, 73, 88, 97, 103]. This positive health-seeking behaviour was also demonstrated in a study by Gadoth et al., where 58 out of 65 women who screened positive for an STI returned for at least one follow-up test of cure [64].

With poor knowledge noted to be an important barrier to testing uptake, satisfactory pre-test counselling is therefore an important facilitator to testing, with provider counselling noted as a reason for satisfaction with some testing services [28, 53]. Conversely, inadequate patient counselling may be detrimental to testing uptake and some studies noted concerns in this regard [50, 60]. For example, Fleming et al. noted that mothers did not always feel fully informed about the tests and were wary of asking staff for more information [43]. Additionally, only 17.5% of pregnant women in a study in dispensaries in rural Kenya had syphilis explained to them during the consultation [49]. Baker et al. also noted that in Uganda, expectant mothers were noted to have poor knowledge for the reasons behind syphilis screening and would avoid testing if there were long waiting times [44]. Furthermore, Nkamba et al. noted that “some providers and clinic administrators stated that it is difficult to provide information to women due to high rates of illiteracy”. They note that suggestions to tackle this included use of images or drawings to supplement counselling [57].

#### General uptake of and trust in health services

Uptake of testing within healthcare environments is also determined by general uptake of health services within communities. This can work both positively and negatively. For example, high levels of access to antenatal care (ANC) by pregnant women are often noted, even in rural environments where accessibility may be difficult [29, 45]. However, delayed attendance at ANC was documented regularly in studies across sub-Saharan Africa [29, 44, 50, 57]. Late diagnosis and treatment may affect the efficacy of treatment in preventing congenital syphilis.

Multiple factors will affect general uptake of health services. However, trust was noted to be an important factor in some studies, including general distrust of the medical system [57], and trust in the organisation providing testing [80]. This may be particularly important for key populations and marginalised groups.

#### Stigma and the influence of partners and peers

Stigma surrounding STI testing was noted to be an important barrier. Nkamba et al. reported that women had fears of being stigmatised by other community members and their partners [57]. As a result, some people did not want to be seen at a health facility. This may affect an individual’s perception of the whole process. When asked about a rapid syphilis testing service set up in Mongolian ANC services, 38.6% of respondents either agreed or strongly agreed that it was felt to be “stressful and less confidential” [53].

Peers could have a direct positive influence or negative influence on testing uptake. Sabido et al. noted that knowledge of people who had already been tested was one of the primary motivators for testing, alongside perceived risk of infection [80]. However, Hall et al. reported that when friends did not accept testing, this sometimes led to an unwillingness to test [87].

Treatment of partners is a key component of STI control to prevent reinfection of the index patient and break the cycle of transmission. Partner resistance to testing and treatment [40, 64], and also a lack of provision for partner notification within clinical settings [28] may be detrimental to STI control at both individual and community levels. Furthermore, barriers to same day treatment for positive results may include a preference by healthcare providers to ensure treatment of both the index case and their partner together at a separate appointment, potentially negating one of the key benefits of point-of-care testing [57].

#### Individual preferences for method of testing

Across different studies, individuals had different preferences for the mode of testing. Generally, there was a preference for fingerprick testing over venepuncture [53, 69, 70, 72, 76, 80], with some specifically mentioning that was because less blood was taken [29, 38, 47]. Other noted benefits included that visibility of the results increased clients trust in them [24, 50] and perceived increased confidentiality through rapid testing [83].

However, barriers to fingerprick testing included pain [38, 51, 76, 79, 80, 82, 87, 92], general fear of fingerprick testing [86], worry about infection from the fingerprick [76, 87] and concerns that testing would affect their sex work [76].

Regarding sampling for CT, NG and TV, a preference for vaginal swabs over urine samples was noted in two studies [68, 101], as well as a preference for self-collection of swabs [68]

#### Context specific factors affecting testing uptake

Multiple studies reported factors associated with uptake of point-of-care testing for STIs, predominantly syphilis [34, 53, 56, 75, 76, 79, 87, 101]. Higher education level [34, 53, 101] and increased age [53, 76, 101] were noted to be associated with higher levels of testing uptake in three studies each. Other factors that were reported as being associated with higher or lower levels of uptake appeared to be much more context-specific, often reported in only one study. This included risk factors such as presence of STI symptoms [79], increased number of sexual partners [87] and different locations for sex work [75, 76, 79].

### Healthcare provision factors

#### Effect on workload, waiting times and clinic flow

Point-of-care testing appeared to have different effects on different existing healthcare systems. Crucially, the ability to test and treat at the same visit meant that less visits were required with reduced time to treatment [24, 28, 36, 38, 42, 47, 53, 61, 73, 74]. Some studies also noted that introduction of testing actually reduced clinic waiting time [24, 50, 73] and allowed more patients to be tested per day [38, 47].

However, other studies reported increased consultation times [35, 71], increased waiting times [53, 65, 97], and disruption to clinic flow [32, 74, 99]. The analytic time was often an issue causing delays [50, 74], with the RPR noted to be particularly time-consuming [23, 42]. Badman et al. found that integration of CT/NG/TV testing into routine practice increased waiting times by 2 h [65]. As discussed above, for clients with pressures on their time, this may have an adverse effect on collection of results and treatment completion. In that study, 11 out of 67 patients with positive results had to leave prior to receiving treatment. The introduction of POCTs also lead to more complex consultations requiring multiple procedures [35, 68, 71, 96] and was often felt to generally increase workload for staff [24, 28, 32, 44–46, 50, 53, 61, 62, 71, 78, 99].

#### Staff knowledge and motivation

Multiple studies noted healthcare provider enthusiasm and satisfaction through offering a same day testing and treatment service, or a desire for point-of-care testing where it was not yet available [24, 50, 53, 57, 60, 74, 93, 99]. It was obviously important for HCWs to have knowledge of the benefits of testing and treatment [28, 57], with benefits derived for both HCWs and clients through learning about new infections [63]. Job satisfaction is also likely to improve if POCTs can provide tangible benefits to care [50, 96]. Importantly, clients valued caring staff [80].

As a result, important barriers to implementation included poor knowledge and awareness amongst HCWs of the burden of STIs, particularly syphilis in pregnancy [23, 26, 44, 45, 57], as well as poor attitudes, low motivation or a lack of commitment to screening [23, 45, 50, 60, 92].

#### Poor documentation and record keeping

Inadequate record keeping was a commonly reported theme, with test results and treatment not consistently recorded [25, 26, 28, 33, 43, 49, 60, 71]. HCWs specifically complained that too much documentation was required [28, 35] and that there was a lack of integration of client records [25]. Where there is no single source to monitor outcomes, such as a central ANC register with all relevant data, monitoring of outcomes becomes more difficult and there is also likely to be duplication of data if recording takes place across multiple registers or records.

#### Confidence in point-of-care testing

Multiple studies noted reduced confidence in point-of-care testing, which has the potential to hinder implementation [55, 61, 69, 70, 76, 80, 82, 83, 85]. For example, although most HCWs interviewed by Marks et al. reported confidence in the point-of-care results, a number of individuals noted that their confidence in the test was reduced as a result of differing results between point-of-care and laboratory testing, as well as previous experiences of discordant results with malaria RDTs [74].

#### Strategies to address worries about change

Although task shifting was noted by some to be a perceived benefit of POCTs [96], some healthcare providers worried about change to existing structures and processes. Laboratory workers worried that POCTs would compromise their authority [32, 38, 61]. However, identification of workplace champions and the explanation the key roles of different providers, such as laboratory workers overseeing quality control, helped to manage these worries and facilitate implementation [61].

### Policy, infrastructure and health system factors

#### Supply chains, stockouts and procurement

Stockouts were a significant issue in the delivery of testing services across a range of settings [23, 24, 26–29, 32, 33, 35, 38, 40, 41, 43–45, 49, 56, 57, 60, 92, 96]. Additionally, there were also accessibility issues with syphilis tests and treatment never available in some clinics [48, 93, 96]. For example, Vani et al. report that syphilis testing in Madhya Pradesh was not offered to clients attending facilities below district level [26].

Different contexts will need different stock management and supply chain solutions. Garcia et al. reported that central procurement of dual HIV/syphilis tests was an improvement on the previous system of clinics buying locally, which resulted in variation in test kits, stockouts, and poor-quality control [61]. Nnko et al. reported that introduction of POCTs for syphilis led to improved supply chains of test kits and penicillin [47].

#### Complex funding pathways

Supply chain management and funding pathways are intimately linked. Therefore, complex funding and ordering pathways may have detrimental effects on stock management [23, 26, 44]. In LMIC, there are often separate, vertical funding structures for different disease programmes [23, 44, 50]. This may result in very uneven funding for different programmes, based on funder preference rather than need. This may also result in “borrowing” of supplies from other programmes with insufficient resources [23]. Other concerns regarding funding include high initial investment costs [64], source of long-term funding [43, 53] and proof of cost-effectiveness [41].

#### Engagement with stakeholders including authorities, policymakers and international partners

Introduction of point-of-care testing at scale is a large undertaking, and requires significant collaboration with relevant stakeholders, with studies noting this as a key to success [31, 38, 61, 75, 92]. Frequent communication and genuine engagement with authorities and policymakers was stated to be crucial, to help instigate policy change [38, 61]. Furthermore, engagement with local stakeholders is also essential to ensure support and buy-in from the community [75].

Unfortunately, some studies noted a lack of support or mentorship from international partners, who were perceived to be more focussed on other conditions such as prevention of mother to child transmission of HIV [44, 50].

#### Integrating into and learning from existing processes

The ability to integrate effectively into existing clinic processes was an important facilitator [37, 50, 53, 68, 97]. This requires the intervention to be flexible and adaptable to different working conditions [50]. Additionally, it is preferable to learn from and improve existing, functioning systems, rather than try and implement an entirely new system through a top-down approach [25]. Otherwise, the consequences may be disruption of existing efficient work processes. Although De Schacht et al. reported an overall increase in coverage of syphilis testing following implementation of POCTs, a significant decline was seen in some health facilities with previously high screening rates [46].

#### Guidelines as facilitators to testing

The inclusion of point-of-care testing into guidelines and protocols was noted to facilitate their use [27, 35, 57]. Conversely, lack of inclusion in guidelines or policy documents, or lack of guidelines entirely were barriers [26, 28, 43, 57].

#### Weak, fragmented health systems

One of the fundamental issues to implementing a new intervention into healthcare systems in LMIC, is that they are often weak, highly fragmented, and have significant infrastructural barriers [27, 50, 57]. This may include water shortages, power cuts and load shedding [57, 67]. This, in conjunction with high patient volumes [50, 57, 99] may in turn may result in poor working conditions for staff [50], exacerbating high staff turnover, staff shortages, and absenteeism [26, 27, 38, 40, 43, 49, 50, 57, 92, 99]. Implementation of a new testing strategy into existing pathways may therefore require additional resources over and above the testing equipment, for successful integration [51]. For wider scale implementation, one must also consider uptake across both public and private facilities, and across different levels of care, where incentives for uptake of testing may differ [32, 34, 60].

### Training, audit, and feedback

#### Provision of high-quality training to all relevant staff

High-quality training was an important facilitator to testing [27, 50, 53, 58, 60, 61, 80]. Difficulties were therefore reported when training was felt to be insufficient [24, 26, 28, 35, 43, 45, 57, 96], not uniform [50, 92], or where training was not provided to all relevant staff [23, 35]. Watson-Jones et al. noted that often only one HCW was trained at each site, which was problematic for leave and illness cover, or if they were transferred before a replacement was trained [23].

Particular aspects of the testing process that HCWs had difficulty with were communicating positive results [40, 92], the added complexities of dual testing [27], and understanding treatment regimens and storage conditions [92]. Additionally, some staff were reported to not follow available guidelines [35, 44].

#### Supervision with refresher or remedial training

Ongoing supervision with focussed refresher or remedial training was identified as important in addressing poor testing practices and maintaining high standards [23, 27, 40, 42, 49, 50]. Garcia et al. noted that daily monitoring and supervision in the first months following introduction of point-of-care syphilis testing into ANC provided the necessary support to incorporate testing into their already busy schedules [61]. Supervision frequency was able to be gradually reduced as HCWs adapted to the new schedule. Similarly, Young et al. noted that HCWs appreciated observation and remedial training to ensure maintenance of skills [50].

#### Quality assurance and control, and accountability

Several studies noted a lack of quality assurance and control. In some settings, this was difficult due to a wide variation in the type of test kits used for screening [26]. However, in other situations there were simply few provisions in place to provide quality assurance and control, and triggers for action, such as fluctuating monthly prevalence rates, were not acted upon [23]. Barriers to the provision of quality assurance and control were similar to barriers to testing itself, including a “lack of trained staff, dedicated time, transport and reporting infrastructure” [24]. In one study where no quality control programme was in place, health workers suggested monthly quality control checks with positive and negative controls, with internal checks provided by laboratory staff [28].

Ongoing monitoring and evaluation of testing programmes may be hampered if STI screening is not a priority within health ministries [26]. This is unfortunate as real-time data is helpful in motivating staff, addressing issues early, and enables accountability [32].

### Reaching new testing environments

#### Outreach services

Point-of-care testing can be an important enabler of testing for marginalised and at-risk populations, for whom access to clinical services is difficult. Mobile testing has been shown to reach higher risk individuals than clinic-based testing [84]. However, testing in outreach scenarios may have different facilitators and barriers to their implementation, that must be considered. For example, studies assessing syphilis and HIV testing in MSM have noted the importance of testing availability in alternative venues such as bars and clubs, and public places like parks [85], as well as environments that were “relaxed and gay-friendly” [83].

This must be balanced however with the difficulty of finding discrete locations within alternative venues [85], concerns about professionalism and quality of testing in non-traditional settings [83] and the need for linkage to care for those with positive results, if treatment is unable to be offered on-site [76, 77, 85].

#### Self and home testing

The availability of user-friendly POCTs allows for the possibility of self- and home-testing [83, 101, 102]. This may allow for increased privacy and confidentiality [101]. Importantly, in a study by Lippman et al., out of 910 eligible women, only one declined to participate because of feeling uncomfortable with self-collection and testing. Important barriers to address include worries about errors when self-testing [101] and ensuring clear instructions to facilitate self-testing and reading results [101, 102].

#### Testing in remote and rural regions

In remote and rural regions, barriers for transport of tests and consumables include large distances, cost and availability of transportation and significant geographical barriers [57, 92]. For example, in regions of the Brazilian Amazon, where air and fluvial transport were the only viable transport options, there was no access to some clinical bases during the dry season [92]. Although point-of-care testing may alleviate some of these barriers, in comparison to laboratory-based testing, rigorous planning is required when delivering testing programmes in remote regions to prevent insufficient consumables in the field preventing testing [95].

Point-of-care testing enables decentralisation of care [40, 50, 74, 92]. In addition to preventing sample loss when sending to a laboratory for testing [41, 42], it also presents an opportunity for testing in areas where previously no testing may have been available, at least without significant travel time [25, 74]. It has also been suggested that point-of-care testing may be particularly beneficial in low-volume testing sites [70], where transport of samples to a central laboratory may not be feasible for either structural or financial reasons.

### Dual testing

#### Dual testing saving time and human resources

Across all papers where dual testing was examined, usually with a combined HIV/syphilis RDT, dual testing was felt to be beneficial in terms of saving both time and human resources [27, 28, 61, 73], as well as reducing the number of blood draws for patients [28, 51, 69, 70, 72, 81]. Barriers to their use identified by Maddox et al. included the need for dual tests to provide cost-savings over and above two single-pathogen tests, as well as the additional procedural steps required to conduct the dual test [27].

The potential benefits of dual treponemal and non-treponemal syphilis testing were also stated across a range of studies, to prevent either the need for confirmatory laboratory testing, or overtreatment [46, 70, 75, 79, 80, 89].

#### Positive synergy of testing for multiple conditions

In addition to increased efficiency at the point of care, dual tests may allow STI tests to benefit from structures in place for HIV testing. For example, linking syphilis testing with HIV testing was felt to provide benefits both in terms of an improved perceived value of syphilis testing given its integration with the routine, well-funded HIV test, as well as preventing stockouts because of stronger HIV supply chains [27].

A positive synergistic effect was also found when implementing integrated packages of separate tests and interventions [46, 61]. For example, Dassah et al. found that pregnant women screened for HIV, or who received intermittent preventative therapy for malaria, were more likely to be screened for syphilis [34].

Conversely, following the introduction of syphilis POCTs into ANC in Zambia, there was a significant increase in ANC attendance, HIV testing, and the number of women receiving antiretroviral prophylaxis [38]. It was suggested this could be due to increased awareness of HIV and syphilis in pregnancy as well as “greater efficiency of integrated services” [38].

## Discussion

The aim of this scoping review was to assess how point-of-care testing for STIs has been implemented into healthcare systems in LMIC and the facilitators and barriers to doing so. This review has revealed a broad, diverse evidence base featuring different study methodologies, different POCTs and target populations, and different approaches to testing. The use of thematic analysis has allowed us to infer seven key themes from this data, that may be helpful in the design of future interventions and the implementation of POCTs into existing clinical services.

Ideal test characteristics generally conformed with the WHO “ASSURED” criteria, a set of target characteristics for POCTs for STIs, whereby they should aim to be affordable, sensitive, specific, user-friendly, rapid, robust, equipment-free, and deliverable in resource-limited settings [105]. Ongoing diagnostics research is necessary to develop POCTs for CT and NG that meet more, if not all these criteria. For example, the binx io CT/NG assay is now available on the market and has an analytic time of only thirty minutes [106], however current cost is likely to be prohibitive to a widescale rollout in LMICs. Importantly, the need for equipment was a barrier to the use of POCTs, and so accurate RDTs are still the most suitable type of test.

The overarching themes “client factors”, “healthcare provision factors”, and “policy, infrastructure and health system factors” mirror the “patient-level”, “facility-level”, and “health system” factors noted to influence the implementation of rapid syphilis and HIV testing in ANC by Swartzendruber et al. [107]. Stockouts, quality assurance, and preference for fingerprick testing over venepuncture were noted by both reviews.

Despite the range of studies included in this review, two sub-themes within “client factors”, namely “individual preferences for method of testing” and “context specific factors affecting testing uptake”, emphasise the need for ongoing research, particularly qualitative studies, at a local level to develop nuanced strategies, suitable for the populations served, and to target sub-populations with low uptake. Context-specific client factors associated with uptake were often variable, only noted in single studies, and did not coalesce to form themes across different environments. As such, this makes extrapolation to other settings difficult. Increased age was noted to be associated with increased syphilis testing uptake in multiple studies, which aligns with the literature, with youth reported to lack knowledge about STIs and available services, as well as experience additional barriers related to acceptability and accessibility of services [108]. Regarding methods of testing, a preference for vaginal swabs over a urine sample was noted in two included studies [68, 101]. This concurs with a previous systematic review, which found a slightly higher preference for vaginal swabs over urine samples [109]. However, again it is of utmost importance to assess the needs of the local population, and to offer multiple options where possible.

Crucially, many of the barriers identified across multiple themes were symptoms of wider health system weakness, including high workload, infrastructural barriers, poor documentation, supply chain issues, complex funding pathways, and inadequate quality assurance. Importantly, POCTs are a useful tool that can ameliorate some problems. However, they are not a “fix-all” and globally we must continue to invest in the fundamentals of healthcare provision, to strive to achieve universal health coverage (UHC), as per the sustainable development goals [110]. Reid et al. reported that health workforce density ranked as the most important element in determining UHC in LMIC [111]. Without investment in developing the capacity and accessibility of the health workforce, POCTs will be unable to fulfil their potential. Furthermore, similarly to the review by Kuupiel et al., stockouts were a significant issue for the provision of point-of-care testing [12]. Robust funding, procurement, and supply chain processes are essential to ensure an uninterrupted supply of consumables for both testing and treatment, to ensure populations receive appropriate care.

This scoping review has revealed some important gaps in the literature, where further primary research is warranted. The evidence base supporting the use of syphilis RDTs is much stronger than for CT, NG, and TV, which is mirrored by the recommendations for the use of syphilis RDTs in ANC by the WHO [112, 113]. As such, more research is required to trial POCTs for CT, NG, and TV, in different settings and with different implementation strategies. Importantly, syphilis and dual HIV/syphilis RDTs have been shown to be cost-effective in settings with varying prevalences [114, 115]. Unfortunately, point-of-care testing for CT and NG is still relatively expensive. In addition to research on cost-effectiveness of CT/NG point-of-care testing, consideration should be made to the development of algorithms to allocate tests to individuals based on their risk of an STI, such as done by Verwijs et al. [97], which may allow conservation of resources. However, a risk prediction tool may be inappropriate if prevalence is high even amongst individuals without risk factors. For example, non-selective testing was felt to be more appropriate for young people in Zimbabwe by Kranzer et al., as risk prediction tools developed were insufficiently sensitive for CT/NG [116].

Additionally, self-testing in LMIC was only trialled in two studies assessing the use of the OSOM Trichomonas Rapid Test [101, 102]. However, the scoping review by Ong et al. notes syphilis self-testing as a potential strategy to expand screening in key populations [117]. Since the initial search, studies in China and Zimbabwe have explored the role of syphilis self-testing in MSM [118, 119], both of which noted the potential of self-testing to complement facility-based testing and facilitate testing of key populations.

This review has several strengths. It was conducted according to Joanna Briggs Institute methodology, with two independent reviewers reviewing papers for study selection, and extracting data. The scale of the review is also advantageous in that it reduced the influence of individual studies, and the plentiful data allowed for the development of rich themes. However, it is not without limitations. Although beneficial in some respects, the sheer number of relevant papers and coding of the data means that some of the nuances in the data may have been lost. There were also some instances where a single research project may have led to more than one publication, such as a clinical trial and a qualitative component, both of which were included because of the broad inclusion criteria [80, 82]. This may add additional weight to certain projects. However, the thematic nature of analysis, and the large number of included studies will have hopefully ameliorated this.

Regarding the search strategy, the approach was broad, and reference lists were also searched for suitable publications. As such, the risk of missing key papers was low. However, as the search was limited to English language articles only, potentially relevant studies may have been missed, which could have introduced bias into our review. Furthermore, the search was performed on 5th June 2020, and so more recent relevant studies will have been published since, of which some are known [118–120]. However, given the high number of included studies, and that saturation was reached, an updated search was not felt to be warranted.

This review has focussed on curable STIs, which excludes viral STIs including HIV, human papillomavirus, herpes simplex virus, hepatitis B, and hepatitis C. The different transmission dynamics, the inability to institute a same day testing and treatment strategy, and different public health considerations, mean it would have been inappropriate to include all these infections in the review. Bacterial vaginosis (BV) was also excluded as it is not an STI, and symptomatic BV would be adequately treated by syndromic management. Screening and treating asymptomatic BV, particularly in pregnancy, is controversial and currently not recommended [121, 122].

Further limitations include that there was no cohesive, objective measure of feasibility, and test uptake was an imperfect surrogate for acceptability. It was not often possible to disentangle acceptability and feasibility, for example if test uptake was not possible due to a stockout. Furthermore, if testing required consent for a research study, potentially with additional questionnaires or follow-up, this may not truly represent uptake in normal circumstances.

## Conclusions

Overall, this review contributes to the current evidence base by offering a cohesive synthesis of a range of studies. Regarding future research and practice, the themes and sub-themes could be used as a basis for a checklist or toolkit when implementing or evaluating the use of POCTs for STIs in LMICs. Crucially, this review emphasises the nuances of testing in different environments, including different workloads, different infrastructure, and different populations. These must be considered when designing interventions, ideally with the meaningful engagement of the clients, healthcare workers, and communities involved. It also demonstrates the critical state of healthcare infrastructure in some LMICs, and the stark contrast with health systems in high income countries. This review will hopefully draw further attention to these severe limiting factors to the provision of routine healthcare in LMICs. Importantly, POCTs are not a panacea and will not be suitable for all environments. In addition to continuing to strive for more equitable access to healthcare globally, we must continue to evaluate the suitability for implementation of POCTs into health systems on their merits and limitations. In particular, more research is required for CT, NG, and TV testing, and self-testing, especially regarding their implementation and cost-effectiveness. As discussed, POCTs have the potential to expand access to testing to new populations, whether by reaching marginalised populations with poor access to formal healthcare, or by being accessible to those living in remote or rural locations. However, centralised, laboratory testing will always be a key component of any national or international testing strategy, and laboratorians must continue to be supported.

## Supplementary Information

**Additional file 1: Appendix S1.** Search strategies.

**Additional file 2: Appendix S2.** Preferred Reporting Items for Systematic Reviews and Meta-analyses Extension for Scoping Reviews (PRISMA-ScR) Checklist.

## Data Availability

Additional data not contained within the manuscript and its Additional files 1, 2 is available from the corresponding author on reasonable request.

## References

[1] Rowley J, Vander Hoorn S, Korenromp E, Low N, Unemo M, Abu-Raddad LJ (2019). Chlamydia, gonorrhoea, trichomoniasis and syphilis: global prevalence and incidence estimates, 2016. Bull World Health Organ.

[2] Farley TA, Cohen DA, Elkins W (2003). Asymptomatic sexually transmitted diseases: the case for screening. Prev Med.

[3] Martin K, Olaru ID, Buwu N, Bandason T, Marks M, Dauya E (2021). Uptake of and factors associated with testing for sexually transmitted infections in community-based settings among youth in Zimbabwe: a mixed-methods study. Lancet Child Adolesc Health.

[4] Peeling RWW, Mabey D (2019). Point-of-care tests to reduce the burden of sexually transmitted infections. Lancet Infect Dis.

[5] Wi TE, Ndowa FJ, Ferreyra C, Kelly-Cirino C, Taylor MM, Toskin I (2019). Diagnosing sexually transmitted infections in resource-constrained settings: challenges and ways forward. J Int AIDS Soc.

[6] Der JB, Grint D, Narh CT, Bonsu F, Grant AD (2020). Where are patients missed in the tuberculosis diagnostic cascade? A prospective cohort study in Ghana. PLoS ONE.

[7] Tang EC, Segura ER, Clark JL, Sanchez J, Lama JR (2015). The syphilis care cascade: tracking the course of care after screening positive among men and transgender women who have sex with men in Lima, Peru. BMJ Open.

[8] Chimbindi N, Bor J, Newell ML, Tanser F, Baltussen R, Hontelez J (2015). Time and money: the true costs of health care utilization for patients receiving “Free” HIV/tuberculosis care and treatment in rural KwaZulu-Natal. J Acquir Immune Defic Syndr.

[9] World Health Organization. Guidelines for the management of symptomatic sexually transmitted infections 2021. Available from: https://www.who.int/publications/i/item/9789240024168. 34370424

[10] Tucker JD, Bu J, Brown LB, Yin YP, Chen XS, Cohen MS (2010). Accelerating worldwide syphilis screening through rapid testing: a systematic review. Lancet Infect Dis.

[11] Phang Romero Casas C, Martyn-St James M, Hamilton J, Marinho DS, Castro R, Harnan S. Rapid diagnostic test for antenatal syphilis screening in low-income and middle-income countries: a systematic review and meta-analysis. BMJ Open. 2018;8(2):e018132. 10.1136/bmjopen-2017-018132. 10.1136/bmjopen-2017-018132 PMC5855314 29467132

[12] Kuupiel D, Bawontuo V, Drain PK, Gwala N, Mashamba-Thompson TP (2019). Supply chain management and accessibility to point-of-care testing in resource-limited settings: a systematic scoping review. BMC Health Serv Res.

[13] Swartzendruber A, Steiner RJ, Adler MR, Kamb ML, Newman LM. Introduction of rapid syphilis testing in antenatal care: a systematic review of the impact on HIV and syphilis testing uptake and coverage. Int J Gynaecol Obstet. 2015;130 Suppl 1(Suppl 1):S15–21. 10.1016/j.ijgo.2015.04.008. 10.1016/j.ijgo.2015.04.008 PMC6799988 26001704

[14] Shahrook S, Mori R, Ochirbat T, Gomi H (2014). Strategies of testing for syphilis during pregnancy. Cochrane Database Syst Rev.

[15] Martin K, Roper T, Vera JH (2021). Point-of-care testing for sexually transmitted infections in low- and middle-income countries: a scoping review protocol. JBI Evid Synth.

[16] Aromataris E, Munn Z (Editors). JBI Manual for Evidence Synthesis. JBI, 2020. Available from https://synthesismanual.jbi.global. 10.46658/JBIMES-20-01.

[17] World Bank. World Bank country and lending groups. Available from: https://datahelpdesk.worldbank.org/knowledgebase/articles/906519-world-bank-country-and-lending-groups.

[18] Cochrane Effective Practice and Organisation of Care (EPOC) Group. LMIC filters. Available from: https://epoc.cochrane.org/lmic-filters.

[19] Young H, Moyes A, de Ste CI, McMillan A (1998). A new recombinant antigen latex agglutination test (Syphilis Fast) for the rapid serological diagnosis of syphilis. Int J STD AIDS.

[20] Herring AJ, Ballard RC, Pope V, Adegbola RA, Changalucha J, Fitzgerald DW, et al. A multi-centre evaluation of nine rapid, point-of-care syphilis tests using archived sera. Sex Transm Infect. 2006;82 Suppl 5(Suppl 5):v7–12. 10.1136/sti.2006.022707. 10.1136/sti.2006.022707 PMC2563911 17118953

[21] Braun V, Clarke V (2006). Using thematic analysis in psychology. Qual Res Psychol.

[22] Saunders B, Sim J, Kingstone T, Baker S, Waterfield J, Bartlam B (2018). Saturation in qualitative research: exploring its conceptualization and operationalization. Qual Quant.

[23] Watson-Jones D, Oliff M, Terris-Prestholt F, Changalucha J, Gumodoka B, Mayaud P (2005). Antenatal syphilis screening in sub-Saharan Africa: lessons learned from Tanzania. Trop Med Int Health.

[24] Ansbro EM, Gill MM, Reynolds J, Shelley KD, Strasser S, Sripipatana T (2015). Introduction of syphilis point-of-care tests, from pilot study to national programme implementation in Zambia: a qualitative study of healthcare workers’ perspectives on testing, training and quality assurance. PLoS ONE.

[25] Dassah ET, Adu-Sarkodie Y, Mayaud P (2015). Estimating the uptake of maternal syphilis screening and other antenatal interventions before and after national rollout of syphilis point-of-care testing in Ghana. Int J Gynecol Obstet.

[26] Vani S, Turlapati PLN, Bhola AK, Singh AK, Shobini R, Gupta RS (2015). Towards elimination of parent-to-child transmission of syphilis in India: a rapid situation review to inform national strategy. WHO South East Asia J Public Health.

[27] Maddox BLP, Wright SS, Namadingo H, Bowen VB, Chipungu GA, Kamb ML (2017). Assessing stakeholder perceptions of the acceptability and feasibility of national scale-up for a dual HIV/syphilis rapid diagnostic test in Malawi. Sex Transm Infect.

[28] Dassah ET, Adu-Sarkodie Y, Mayaud P (2018). Rollout of rapid point of care tests for antenatal syphilis screening in Ghana: healthcare provider perspectives and experiences. BMC Health Serv Res.

[29] Kanyangarara M, Walker N, Boerma T (2018). Gaps in the implementation of antenatal syphilis detection and treatment in health facilities across sub-Saharan Africa. PLoS ONE.

[30] Olugbenga I, Taiwo O, Laverty M, Ngige E, Anyaike C, Bakare R (2018). Clinic-based evaluation study of the diagnostic accuracy of a dual rapid test for the screening of HIV and syphilis in pregnant women in Nigeria. PLoS ONE.

[31] Garcia SG, Tinajeros F, Revollo R, Yam EA, Richmond K, Diaz-Olavarrieta C (2007). Demonstrating public health at work: a demonstration project of congenital syphilis prevention efforts in Bolivia. Sex Transm Dis.

[32] Severe L, Benoit D, Zhou XK, Pape JW, Peeling RW, Fitzgerald DW (2013). Rapid-testing technology and systems improvement for the elimination of congenital syphilis in Haiti: overcoming the “Technology to Systems Gap”. J Sex Transm Dis.

[33] Bonawitz RE, Duncan J, Hammond E, Hamomba L, Nambule J, Sambambi K (2015). Assessment of the impact of rapid syphilis tests on syphilis screening and treatment of pregnant women in Zambia. Int J Gynecol Obstet.

[34] Dassah ET, Adu-Sarkodie Y, Mayaud P (2015). Factors associated with failure to screen for syphilis during antenatal care in Ghana: a case control study. BMC Infect Dis.

[35] Bocoum FY, Tarnagda G, Bationo F, Savadogo JR, Nacro S, Kouanda S (2017). Introducing onsite antenatal syphilis screening in Burkina Faso: implementation and evaluation of a feasibility intervention tailored to a local context. BMC Health Serv Res.

[36] Wang Q, Chan P, Newman LM, Dou L, Wang X, Qiao Y (2018). Acceptability and feasibility of dual HIV and syphilis point-of-care testing for early detection of infection among pregnant women in China: a prospective study. BMJ Open.

[37] Strasser S, Bitarakwate E, Gill M, Hoffman HJ, Musana O, Phiri A (2012). Introduction of rapid syphilis testing within prevention of mother-to-child transmission of HIV programs in Uganda and Zambia: a field acceptability and feasibility study. J Acquir Immune Defic Syndr.

[38] Mabey DC, Sollis KA, Kelly HA, Benzaken AS, Bitarakwate E, Changalucha J (2012). Point-of-care tests to strengthen health systems and save newborn lives: the case of syphilis. PLoS Med.

[39] Wilkinson D, Sach M (1998). Improved treatment of syphilis among pregnant women through on-site testing: an intervention study in rural South Africa. Trans R Soc Trop Med Hyg.

[40] Smith A, Sabidó M, Camey E, Batres A, Casabona J (2015). Lessons learned from integrating simultaneous triple point-of-care screening for syphilis, hepatitis B, and HIV in prenatal services through rural outreach teams in Guatemala. Int J Gynecol Obstet.

[41] Myer L, Wilkinson D, Lombard C, Zuma K, Rotchford K, Karim SS (2003). Impact of on-site testing for maternal syphilis on treatment delays, treatment rates, and perinatal mortality in rural South Africa: a randomised controlled trial. Sex Transm Infect.

[42] Bronzan RN, Mwesigwa-Kayongo DC, Narkunas D, Schmid GP, Neilsen GA, Ballard RC (2007). On-site rapid antenatal syphilis screening with an immunochromatographic strip improves case detection and treatment in rural South African clinics. Sex Transm Dis.

[43] Fleming E, Oremo J, O'Connor K, Odhiambo A, Ye T, Oswago S (2013). The impact of integration of rapid syphilis testing during routine antenatal services in rural Kenya. J Sex Transm Dis.

[44] Baker U, Okuga M, Waiswa P, Manzi F, Peterson S, Hanson C (2015). Bottlenecks in the implementation of essential screening tests in antenatal care: syphilis, HIV, and anemia testing in rural Tanzania and Uganda. Int J Gynaecol Obstet.

[45] Baker U, Peterson S, Marchant T, Mbaruku G, Temu S, Manzi F (2015). Identifying implementation bottlenecks for maternal and newborn health interventions in rural districts of the United Republic of Tanzania. Bull World Health Organ.

[46] De Schacht C, Lucas C, Sitoe N, Machekano R, Chongo P, Temmerman M (2015). Implementation of point-of-care diagnostics leads to variable uptake of syphilis, anemia and CD4+ T-cell count testing in rural maternal and child health clinics. PLoS ONE.

[47] Nnko S, Changalucha J, Mosha J, Bunga C, Wamoyi J, Peeling R (2016). Perceptions, attitude and uptake of rapid syphilis testing services in antenatal clinics in North-Western Tanzania. Health Policy Plan.

[48] Kuupiel D, Tlou B, Bawontuo V, Mashamba-Thompson TP (2019). Accessibility of pregnancy-related point-of-care diagnostic tests for maternal healthcare in rural primary healthcare facilities in northern Ghana: a cross-sectional survey. Heliyon.

[49] Young N, Taegtmeyer M, Aol G, Bigogo GM, Phillips-Howard PA, Hill J (2018). Integrated point-of-care testing (POCT) of HIV, syphilis, malaria and anaemia in antenatal clinics in western Kenya: a longitudinal implementation study. PLoS ONE.

[50] Young N, Achieng F, Desai M, Phillips-Howard P, Hill J, Aol G (2019). Integrated point-of-care testing (POCT) for HIV, syphilis, malaria and anaemia at antenatal facilities in western Kenya: a qualitative study exploring end-users’ perspectives of appropriateness, acceptability and feasibility. BMC Health Serv Res.

[51] Pai NP, Kurji J, Singam A, Barick R, Jafari Y, Klein MB (2012). Simultaneous triple point-of-care testing for HIV, syphilis and hepatitis B virus to prevent mother-to-child transmission in India. Int J STD AIDS.

[52] Bique Osman N, Challis K, Folgosa E, Cotiro M, Bergstrom S (2000). An intervention study to reduce adverse pregnancy outcomes as a result of syphilis in Mozambique. Sex Transm Infect.

[53] Munkhuu B, Liabsuetrakul T, McNeil E, Janchiv R (2009). Feasibility of one-stop antenatal syphilis screening in Ulaanbaatar, Mongolia: women and providers perspectives. Southeast Asian J Trop Med Public Health.

[54] Munkhuu B, Liabsuetrakul T, Chongsuvivatwong V, McNeil E, Janchiv R (2009). One-stop service for antenatal syphilis screening and prevention of congenital syphilis in Ulaanbaatar, Mongolia: a cluster randomized trial. Sex Transm Dis.

[55] Gaitán-Duarte HG, Gonzalez-Gordon LM, Ángel-Müller E, Rincón C, Newman L, Laverty M (2016). Comparative effectiveness of single and dual rapid diagnostic tests for syphilis and HIV in antenatal care services in Colombia. Rev Panam Salud Publica.

[56] Berrueta M, Cafferata ML, Mwenechanya M, Mukadi DN, Althabe F, Bergel E (2017). Syphilis screening and treatment in pregnant women in Kinshasa, Democratic Republic of the Congo and in Lusaka, Zambia: a cross-sectional study. Gates Open Res.

[57] Nkamba D, Mwenechanya M, Kilonga AM, Cafferata ML, Berrueta AM, Mazzoni A (2017). Barriers and facilitators to the implementation of antenatal syphilis screening and treatment for the prevention of congenital syphilis in the Democratic Republic of Congo and Zambia: results of qualitative formative research. BMC Health Serv Res.

[58] Althabe F, Chomba E, Tshefu AK, Banda E, Belizan M, Bergel E (2019). A multifaceted intervention to improve syphilis screening and treatment in pregnant women in Kinshasa, Democratic Republic of the Congo and in Lusaka, Zambia: a cluster randomised controlled trial. Lancet Glob Health.

[59] Kasaro MP, Bosomprah S, Taylor MM, Sindano N, Phiri C, Tambatamba B (2019). Field performance evaluation of dual rapid HIV and syphilis tests in three antenatal care clinics in Zambia. Int J STD AIDS.

[60] Tinajeros F, Ares LR, Elías V, Reveiz L, Sánchez F, Mejía M (2017). Health-worker barriers to syphilis screening in pregnant women in Bolivia’s Los Andes network. Pan Am J Public Health.

[61] Garcia PJ, Carcamo CP, Chiappe M, Valderrama M, La Rosa S, Holmes KK (2013). rapid syphilis tests as catalysts for health systems strengthening: a case study from Peru. PLoS ONE.

[62] Bocoum FY, Ouedraogo H, Tarnagda G, Kiba A, Tiendrebeogo S, Bationo F (2015). Evaluation of the diagnostic performance and operational characteristics of four rapid immunochromatographic syphilis tests in Burkina Faso. Afr Health Sci.

[63] Pant Pai N, Daher J, Prashanth HR, Shetty A, Sahni RD, Kannangai R (2019). Will an innovative connected AideSmart! App-based multiplex, point-of-care screening strategy for HIV and related coinfections affect timely quality antenatal screening of rural Indian women? Results from a cross-sectional study in India. Sex Transm Infect.

[64] Gadoth A, Shannon CL, Hoff NA, Mvumbi G, Musene K, Okitolonda-Wemakoy E (2020). Prenatal chlamydial, gonococcal, and trichomonal screening in the Democratic Republic of Congo for case detection and management. Int J STD AIDS.

[65] Badman SG, Vallely LM, Toliman P, Kariwiga G, Lote B, Pomat W (2016). A novel point-of-care testing strategy for sexually transmitted infections among pregnant women in high-burden settings: results of a feasibility study in Papua New Guinea. BMC Infect Dis.

[66] Wynn AM. Evaluating routine testing and treatment for sexually transmitted infections among pregnant women in southern Africa [Ph.D.]. Ann Arbor: University of California, Los Angeles; 2017.

[67] Wynn A, Ramogola-Masire D, Gaolebale P, Moshashane N, Agatha Offorjebe O, Arena K (2016). Acceptability and feasibility of sexually transmitted infection testing and treatment among pregnant women in Gaborone, Botswana, 2015. Biomed Res Int.

[68] Morikawa E, Mudau M, Olivier D, de Vos L, Joseph Davey D, Price C (2018). Acceptability and feasibility of integrating point-of-care diagnostic testing of sexually transmitted infections into a South African Antenatal Care Program for HIV-infected pregnant women. Infect Dis Obstet Gynecol.

[69] Bristow CC, Lee S, Severe L, Pape JW, Javanbakht M, Comulada WS (2017). Attributes of diagnostic tests to increase uptake of dual testing for syphilis and HIV in Port-au-Prince, Haiti. Int J STD AIDS.

[70] Laksanasopin T, Guo TW, Nayak S, Sridhara AA, Xie S, Olowookere OO (2015). A smartphone dongle for diagnosis of infectious diseases at the point of care. Sci Transl Med.

[71] Fehler HG, Ballard RC (1998). Pilot study to evaluate the feasibility of on-site RPR screening at antenatal and dedicated sexually transmitted disease clinics in South Africa. South Afr J Epidemiol Infect.

[72] Withers K, Bristow C, Nguyen M, Stafylis C, Giang LM, Klausner JD (2019). A field evaluation of a rapid dual immunoassay for human immunodeficiency virus and syphilis antibodies, Hanoi, Vietnam. Int J STD AIDS.

[73] Flores EC, Lluque ME, Chiappe M, Lino R, Bayer AM (2015). Operations research study to implement HIV and syphilis point-of-care tests and assess client perceptions in a marginalised area of Lima, Peru. Int J STD AIDS.

[74] Marks M, Esau T, Asugeni R, Harrington R, Diau J, Toloka H (2018). Point-of-care tests for syphilis and yaws in a low-income setting—a qualitative study of healthcare worker and patient experiences. PLoS Negl Trop Dis.

[75] Gupte S, Daly C, Agarwal V, Gaikwad SB, George B (2011). Introduction of rapid tests for large-scale syphilis screening among female, male, and transgender sex workers in Mumbai, India. Sex Transm Dis.

[76] Chen XS, Yin YP, Shen C, Liu GG, Zhu ZJ, Wei WH (2012). Rapid syphilis testing uptake for female sex workers at sex venues in southern China: implications for expanding syphilis screening. PLoS ONE.

[77] Campos PE, Buffardi AL, Chiappe M, Buendia C, Garcia PJ, Carcamo CP (2006). Utility of the Determine Syphilis TP rapid test in commercial sex venues in Peru. Sex Transm Infect.

[78] Parthasarathy MR, Narayanan P, Das A, Gurung A, Prabhakar P, Wi T (2013). Integrating syphilis screening in a large-scale HIV prevention program for key populations: the Avahan experience from India. J Infect Dev Ctries.

[79] Mishra S, Naik B, Venugopal B, Kudur P, Washington R, Becker M (2010). Syphilis screening among female sex workers in Bangalore, India: comparison of point-of-care testing and traditional serological approaches. Sex Transm Infect.

[80] Sabidó M, Benzaken AS, de-Andrade-Rodrigues EJ, Mayaud P, Sabidó M, Benzaken AS, et al. Rapid point-of-care diagnostic test for syphilis in high-risk populations, Manaus, Brazil. Emerg Infect Dis. 2009;15(4):647–9. 10.3201/eid1504.081293. 10.3201/eid1504.081293 PMC2671407 19331762

[81] Bristow CC, Kojima N, Lee SJ, Leon SR, Ramos LB, Konda KA (2018). HIV and syphilis testing preferences among men who have sex with men and among transgender women in Lima, Peru. PLoS ONE.

[82] Benzaken AS, Sabido M, Galban EG, Pedroza V, Vasquez F, Araujo A (2008). Field evaluation of the performance and testing costs of a rapid point-of-care test for syphilis in a red-light district of Manaus, Brazil. Sex Transm Infect.

[83] Bien CH, Muessig KE, Lee R, Lo EJ, Yang LG, Yang B (2015). HIV and syphilis testing preferences among men who have sex with men in South China: a qualitative analysis to inform sexual health services. PLoS ONE.

[84] Lipsitz MC, Segura ER, Castro JL, Smith E, Medrano C, Clark JL (2014). Bringing testing to the people—benefits of mobile unit HIV/syphilis testing in Lima, Peru, 2007–2009. Int J STD AIDS.

[85] Allan-Blitz L-T, Herrera MC, Calvo GM, Vargas SK, Caceres CF, Klausner JD (2019). Venue-based HIV-testing: an effective screening strategy for high-risk populations in Lima, Peru. AIDS Behav.

[86] Pinto VM, Tancredi MV, De Alencar HD, Camolesi E, Holcman MM, Grecco JP (2014). Prevalence of syphilis and associated factors in homeless people of Sao Paulo, Brazil, using a rapid test. Rev Bras Epidemiol.

[87] Hall BJ, Yang X, Huang L, Yi G, Chan EWW, Tucker JD (2020). Barriers and facilitators of rapid HIV and syphilis testing uptake among Filipino transnational migrants in China. AIDS Behav.

[88] Benzaken AS, Garcia EG, Sardinha JCG, Dutra JC, Peeling R (2007). Rapid tests for diagnosing syphilis: validation in an STD clinic in the Amazon Region, Brazil. Cadernos de Saude Publica.

[89] Gallo Vaulet L, Morando N, Casco R, Melgar A, Nacher S, Rodriguez Fermepin M (2018). Evaluation of the utility of a rapid test for syphilis at a sexually transmitted disease clinic in Buenos Aires, Argentina. Sci Rep.

[90] Ribeiro LVDC, Sabido M, Galban E, De Oliveira Guerra JA, Mabey D, Peeling RW (2015). Home-based counseling and testing for HIV and syphilis—an evaluation of acceptability and quality control, in remote Amazonas State, Brazil. Sex Transm Infect.

[91] Mark J, Kinuthia J, Roxby AC, Krakowiak D, Osoti A, Richardson BA (2017). Uptake of home-based syphilis and human immunodeficiency virus testing among male partners of pregnant women in Western Kenya. Sex Transm Dis.

[92] Ruffinen CZ, Sabidó M, Díaz-Bermúdez XP, Lacerda M, Mabey D, Peeling RW (2015). Point-of-care screening for syphilis and HIV in the borderlands: challenges in implementation in the Brazilian Amazon. BMC Health Serv Res.

[93] Mashamba-Thompson TP, Sartorius B, Drain PK (2018). Operational assessment of point-of-care diagnostics in rural primary healthcare clinics of KwaZulu-Natal, South Africa: a cross-sectional survey. BMC Health Serv Res.

[94] Smit PW, Mabey D, Changalucha J, Mngara J, Clark B, Andreasen A (2013). The trade-off between accuracy and accessibility of syphilis screening assays. PLoS ONE.

[95] West B, Walraven G, Morison L, Brouwers J, Bailey R (2002). Performance of the rapid plasma reagin and the rapid syphilis screening tests in the diagnosis of syphilis in field conditions in rural Africa. Sex Transm Infect.

[96] Parkes-Ratanshi R, Kikonyogo R, Hsieh Y-H, Nakku-Joloba E, Manabe YC, Gaydos CA (2019). Point-of-care diagnostics: needs of African health care workers and their role combating global antimicrobial resistance. Int J STD AIDS.

[97] Verwijs MC, Agaba SK, Sumanyi J-C, Umulisa MM, Mwambarangwe L, Musengamana V (2019). Targeted point-of-care testing compared with syndromic management of urogenital infections in women (WISH): a cross-sectional screening and diagnostic accuracy study. Lancet Infect Dis.

[98] Garrett NJ, Osman F, Maharaj B, Naicker N, Gibbs A, Norman E (2018). Beyond syndromic management: opportunities for diagnosis-based treatment of sexually transmitted infections in low- and middle-income countries. PLoS ONE.

[99] Stime KJ, Garrett N, Sookrajh Y, Dorward J, Dlamini N, Olowolagba A (2018). Clinic flow for STI, HIV, and TB patients in an urban infectious disease clinic offering point-of-care testing services in Durban, South Africa. BMC Health Serv Res.

[100] Badman SG, Willie B, Narokobi R, Gabuzzi J, Pekon S, Amos-Kuma A (2019). A diagnostic evaluation of a molecular assay used for testing and treating anorectal chlamydia and gonorrhoea infections at the point-of-care in Papua New Guinea. Clin Microbiol Infect.

[101] Jones HE, Altini L, de Kock A, Young T, van de Wijgert JH (2007). Home-based versus clinic-based self-sampling and testing for sexually transmitted infections in Gugulethu, South Africa: randomised controlled trial. Sex Transm Infect.

[102] Lippman SA, Jones HE, Luppi CG, Pinho AA, Veras MA, van de Wijgert JH (2007). Home-based self-sampling and self-testing for sexually transmitted infections: acceptable and feasible alternatives to provider-based screening in low-income women in São Paulo, Brazil. Sex Transm Dis.

[103] Benzaken AS, Galban EG, Antunes W, Dutra JC, Peeling RW, Mabey D (2006). Diagnosis of gonococcal infection in high risk women using a rapid test. Sex Transm Infect.

[104] Yin YP, Peeling RW, Chen XS, Gong KL, Zhou H, Gu WM (2006). Clinic-based evaluation of Clearview Chlamydia MF for detection of *Chlamydia trachomatis* in vaginal and cervical specimens from women at high risk in China. Sex Transm Infect.

[105] Peeling RW, Holmes KK, Mabey D, Ronald A. Rapid tests for sexually transmitted infections (STIs): the way forward. Sex Transm Infect. 2006;82 Suppl 5(Suppl 5):v1–6. 10.1136/sti.2006.024265. 10.1136/sti.2006.024265 PMC2563912 17151023

[106] Van Der Pol B, Gaydos CA (2021). A profile of the binx health io® molecular point-of-care test for chlamydia and gonorrhea in women and men. Expert Rev Mol Diagn.

[107] Swartzendruber A, Steiner RJ, Adler MR, Kamb ML, Newman LM. Introduction of rapid syphilis testing in antenatal care: a systematic review of the impact on HIV and syphilis testing uptake and coverage. Int J Gynaecol Obstet. 2015;130 Suppl 1(Suppl 1):S15–21. 10.1016/j.ijgo.2015.04.008. 10.1016/j.ijgo.2015.04.008 PMC6799988 26001704

[108] Newton-Levinson A, Leichliter JS, Chandra-Mouli V (2016). Sexually transmitted infection services for adolescents and youth in low- and middle-income countries: perceived and experienced barriers to accessing care. J Adolesc Health.

[109] Paudyal P, Llewellyn C, Lau J, Mahmud M, Smith H (2015). Obtaining self-samples to diagnose curable sexually transmitted infections: a systematic review of patients’ experiences. PLoS ONE.

[110] Stenberg K, Hanssen O, Edejer TT, Bertram M, Brindley C, Meshreky A (2017). Financing transformative health systems towards achievement of the health Sustainable Development Goals: a model for projected resource needs in 67 low-income and middle-income countries. Lancet Glob Health.

[111] Reid M, Gupta R, Roberts G, Goosby E, Wesson P (2020). Achieving Universal Health Coverage (UHC): dominance analysis across 183 countries highlights importance of strengthening health workforce. PLoS ONE.

[112] World Health Organization. Prequalified in vitro diagnostics. Available from: https://extranet.who.int/pqweb/vitro-diagnostics/vitro-diagnostics-lists.

[113] World Health Organization. Dual HIV/Syphilis rapid diagnostic tests can be used as the first test in antenatal care 2019. Available from: https://www.who.int/publications/i/item/WHO-CDS-HIV-19.38.

[114] Mallma P, Garcia P, Carcamo C, Torres-Rueda S, Peeling R, Mabey D (2016). Rapid syphilis testing is cost-effective even in low-prevalence settings: the CISNE-PERU experience. PLoS ONE.

[115] Rodriguez PJ, Roberts DA, Meisner J, Sharma M, Owiredu MN, Gomez B (2021). Cost-effectiveness of dual maternal HIV and syphilis testing strategies in high and low HIV prevalence countries: a modelling study. Lancet Glob Health.

[116] Kranzer K, Simms V, Dauya E, Olaru ID, Dziva Chikwari C, Martin K (2021). Identifying youth at high risk for sexually transmitted infections in community-based settings using a risk prediction tool: a validation study. BMC Infect Dis.

[117] Ong JJ, Fu H, Smith MK, Tucker JD (2018). Expanding syphilis testing: a scoping review of syphilis testing interventions among key populations. Expert Rev Anti Infect Ther.

[118] Wang C, Cheng W, Li C, Tang W, Ong JJ, Smith MK (2020). Syphilis self-testing: a nationwide pragmatic study among men who have sex with men in China. Clin Infect Dis.

[119] Sri-Pathmanathan C, Nhamo D, Mamvuto T, Chapwanya G, Terris-Prestholt F, Mahaka I (2021). Syphilis self-testing to expand test uptake among men who have sex with men: a theoretically informed mixed methods study in Zimbabwe. Sex Transm Infect.

[120] Martin K, Dziva Chikwari C, Mackworth-Young CRS, Chisenga M, Bandason T, Dauya E (2022). “It was difficult to offer same day results”: evaluation of community-based point-of-care testing for sexually transmitted infections among youth using the GeneXpert platform in Zimbabwe. BMC Health Serv Res.

[121] Guise JM, Mahon SM, Aickin M, Helfand M, Peipert JF, Westhoff C (2001). Screening for bacterial vaginosis in pregnancy. Am J Prev Med.

[122] Okun N, Gronau KA, Hannah ME (2005). Antibiotics for bacterial vaginosis or *Trichomonas vaginalis* in pregnancy: a systematic review. Obstet Gynecol.

